# Source identification of western Oregon Douglas-fir wood cores using mass spectrometry and random forest classification^1^

**DOI:** 10.3732/apps.1600158

**Published:** 2017-05-12

**Authors:** Kristen Finch, Edgard Espinoza, F. Andrew Jones, Richard Cronn

**Affiliations:** 2 Department of Botany and Plant Pathology, Oregon State University, Corvallis, Oregon 97331 USA; 3 National Fish and Wildlife Forensic Laboratory, Ashland, Oregon 97520 USA; 4 Smithsonian Tropical Research Institute, Balboa, Ancon, Republic of Panama; 5 USDA Forest Service Pacific Northwest Research Station, Corvallis, Oregon 97331 USA

**Keywords:** DART-TOFMS, Douglas-fir, metabolites, provenance, *Pseudotsuga*, wood identification

## Abstract

**Premise of the study:**

We investigated whether wood metabolite profiles from direct analysis in real time (time-of-flight) mass spectrometry (DART-TOFMS) could be used to determine the geographic origin of Douglas-fir wood cores originating from two regions in western Oregon, USA.

**Methods:**

Three annual ring mass spectra were obtained from 188 adult Douglas-fir trees, and these were analyzed using random forest models to determine whether samples could be classified to geographic origin, growth year, or growth year and geographic origin. Specific wood molecules that contributed to geographic discrimination were identified.

**Results:**

Douglas-fir mass spectra could be differentiated into two geographic classes with an accuracy between 70% and 76%. Classification models could not accurately classify sample mass spectra based on growth year. Thirty-two molecules were identified as key for classifying western Oregon Douglas-fir wood cores to geographic origin.

**Discussion:**

DART-TOFMS is capable of detecting minute but regionally informative differences in wood molecules over a small geographic scale, and these differences made it possible to predict the geographic origin of Douglas-fir wood with moderate accuracy. Studies involving DART-TOFMS, alone and in combination with other technologies, will be relevant for identifying the geographic origin of illegally harvested wood.

The Convention on International Trade in Endangered Species of Wild Fauna and Flora (CITES) and the U.S. Lacey Act provide partial or full protection for species that are at risk of over-exploitation via harvest and trade (Lancaster and Espinoza, 2012a; Eberhardt, 2013). The U.S. Lacey Act requires the scientific name, common name, and geographic source to accompany imported wood or finished wood products (Eberhardt, 2013). Despite the substantial risk of penalties and forfeitures, Lacey Act declarations are frequently unreliable and inaccurate due to misidentification, allowing for ∼US$10–15 billion to be lost by governments and businesses globally (Elias, 2012). Accountability for harvest and trade in CITES-protected species requires taxonomic and geographic verification (Dormontt et al., 2015).

Anatomical wood identification relies on morphological characters that range from the simple and macroscopic (e.g., color, weight, and scent) to the complex and microscopic, such as the distribution of resin canals or vessels, and the arrangement of parenchyma and ray cells in wood (Hoadley, 1990). Microscopic examination of wood can typically provide an identification to the level of species, but the wood of closely related species is often nearly identical, and specimens may be incorrectly identified as the wrong taxon, even to the level of family (Wheeler and Baas, 1998). Wood identification resources are digitally available, interactive, and provide macroscopic and microscopic detail for thousands of species (Wheeler et al., 1989; Gasson et al., 2011; Wheeler, 2011). These resources provide a valuable starting point for the identification of protected tree species; however, given the taxonomic diversity and volume of international wood commerce, wood identification based on anatomy is limited by insufficient expertise. Additionally, anatomical verification is time-consuming when shipments contain numerous logs, boards, composites, or finished items such as furniture and musical instruments (Dormontt et al., 2015; McClure et al., 2015). With the high demand for wood and wood products, a taxonomically accurate and rapid method for wood identification is critical for the enforcement of laws regarding the harvest of trees and the trade of wood and wood products.

An even greater challenge than wood taxonomic identification is determining the geographic origin of a wood specimen. It is nearly impossible to identify the geographic origin of a log based on anatomy alone, even from microscopic features (Gasson, 2011). While it is prohibited to harvest some tree species entirely, others (e.g., Spanish cedar [*Cedrela odorata* L.] and Mongolian oak [*Quercus mongolica* Fisch. ex Turcz.]) are legal to harvest across only a limited portion of their natural distribution (Zyryanova et al., 2005; Pennington and Muellner, 2010; Reboredo, 2013). To combat illegal logging and provide supply management tools for legal timber trade, methods for precise identification of wood to geographic provenance are also needed.

Mass spectrometry–based chemical or metabolite screening of wood via direct analysis in real time (time-of-flight) mass spectrometry (DART-TOFMS) has been proposed as a rapid screening tool for wood identification that shows considerable promise for agencies responsible for enforcing international trade regulations (e.g., U.S. Lacey Act of 2008, the European Union Timber Regulation of 2010, CITES; Espinoza et al., 2015; Musah et al., 2015). DART-TOFMS provides an instantaneous small molecule profile for solid samples in an open-air environment, removing the labor-intensive requirement of material preparation in chemical solvent and the potential for sample preparation biases (Cody et al., 2005; Cody, 2013). Differentiation provided by DART-TOFMS metabolite profiles has been used to discriminate wood from many closely related tree species (Cody et al., 2012; Lancaster and Espinoza, 2012a, 2012b; Espinoza et al., 2014, 2015). Due to rapid sample preparation (i.e., less than one minute per sample) and the classification accuracy of this method, DART-TOFMS is now used by the U.S. Fish and Wildlife Service to identify CITES-listed species in wood forensics cases, especially when anatomical identification is not possible (Lancaster and Espinoza, 2012a; Espinoza et al., 2014; McClure et al., 2015).

Although DART-TOFMS is increasingly used to differentiate wood among species, little is known about the ability of DART-TOFMS to discriminate geographic provenances of wood derived from a single species. Local environmental conditions and genetic differences can affect molecule biosynthesis in plants (McGarvey and Croteau, 1995; Litvak et al., 2002; Huber and Bohlmann, 2004; Schnitzler et al., 2004; Huber et al., 2005a, 2005b; Robinson et al., 2007; Loreto and Schnitzler, 2010), and these may impart a signal that allows for identification of different geographic sources of conspecific samples. For example, DART-TOFMS has been used to discriminate fresh herbaceous material from roots of *Angelica gigas* Nakai originating from Korea or China (Kim et al., 2015), and also to discriminate cultivated and wild sources of *Aquilaria* Lam. spp. wood specimens (Espinoza et al., 2014). These studies tested the ability of DART-TOFMS to discriminate differences at a large spatial scale (e.g., >500 km; Kim et al., 2015), but they did not directly address the ability of DART-TOFMS data to resolve fine-scale intraspecific provenances.

Here, we investigated fine-scale variation in wood chemistry to evaluate the potential for identifying the geographic origin of wood based on DART-TOFMS spectra. We screened wood metabolite profiles from wood core samples of Douglas-fir (*Pseudotsuga menziesii* (Mirb.) Franco var. *menziesii*) across a narrowly defined geographic region (distances <100 km) in the North American Pacific Northwest. Douglas-fir is a widespread, economically important tree in this region (Howe et al., 2013). Given its value, Douglas-fir is an attractive target for poaching in national forests and parks (Koehler, 2013). However, the value of Douglas-fir in this context is as an experimental system for testing technologies to reveal fine-scale geographic variation in the features used in forensic wood identification—wood chemistry, genetic markers, or stable isotopes. Spatial variation in wood chemistry can be influenced by genetics and local environmental variation (Huber et al., 2005a, 2005b; Robinson et al., 2007). Although few Douglas-fir wood molecules have been fully described, the wood of Douglas-fir is rich in secondary metabolites or molecules that likely function as growth hormones and defense molecules (Schnitzler et al., 2004; Loreto and Schnitzler, 2010). Due to the dominance of Douglas-fir across a wide array of environments and heterogeneous landscapes in western Oregon (Hermann and Lavender, 1990; Ohmann and Spies, 1998) and its characteristic high levels of phenotypic and genetic variability (St. Clair et al., 2005; Eckert et al., 2009; Krutovsky et al., 2009; Howe et al., 2013), a relationship between geography and molecular composition and abundance is possible, regardless of whether genetics or environmental conditions are responsible for wood chemical variation.

Our specific objective was to determine if DART-TOFMS wood metabolite spectra could be used to differentiate Douglas-fir wood cores from the Oregon Coast Range and Oregon Cascade Range. These two mountain ranges run parallel to the Pacific Ocean and show strong environmental gradients in temperature and precipitation over small geographic distances (∼35–100 km; Ohmann and Spies, 1998; Law et al., 2004). Douglas-fir is continuously distributed across these mountain ranges, and previous genetic analysis shows that the intervening valley is a weak barrier to historical migration and gene flow (Krutovsky et al., 2009). The combination of continuous tree distribution and small geographic scale is relevant to many questions in illegal logging, such as wood theft from specific parts of a larger native range, or from specific administrative units such as reserves or national parks. For this study, we collected wood increment cores from 188 Douglas-fir trees, with approximately equal sampling of the Coast Range and Cascade Range. Sections of dried wood from the 1986–1988 growing seasons were dissected and individually analyzed by DART-TOFMS to obtain sample mass spectra for each tree ring and averaged mass spectra for individual trees over three years. This sampling design allowed us to address two specific questions: (1) Can wood from Douglas-fir trees originating in the Oregon Coast and Cascade ranges be accurately classified to geographic source using their DART-TOFMS metabolite profiles, and if so, which molecules allow for the discrimination of regional sources of wood, and (2) What is the magnitude of interannual variation in wood metabolic molecules relative to that of geographic variation?

## MATERIALS AND METHODS

### Samples

**Fig. 1.**
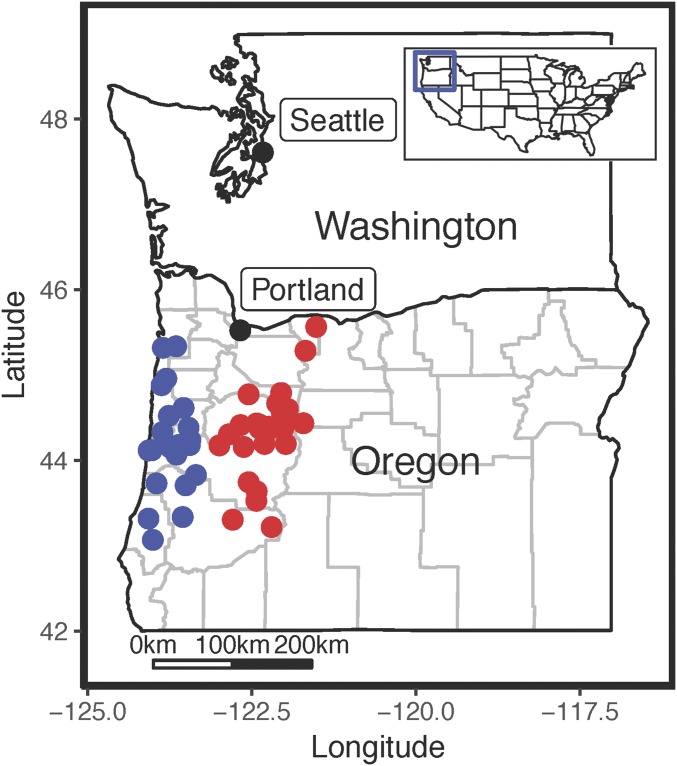
Map of sampled region in western Oregon, USA. Dots show the site of sampled trees, with Cascade Range samples in red and Coast Range samples in blue.

We collected 5.15-mm-diameter wood cores from 188 adult Douglas-fir trees in western Oregon between June and August 2015. We chose sample locations based upon previous studies that characterized the geographic distribution of genetic variation in the species (St. Clair et al., 2005; Krutovsky et al., 2009). We focused our efforts in two geographically distinct mountain ranges in western Oregon, with 23 sampling locations from the Coast Range (bounded by 43.1–45.5°N and 123.5–124.0°W) and 25 sampling locations from the Cascade Range (bounded by 43.1–45.6°N and 121.5–122.7°W). A map showing sampling locations and known source classifications is provided in Fig. 1 (see Appendix 1 for GPS coordinates). At each sampling location, we opportunistically selected two to six trees for a total of 85 trees from the Coast region and 103 trees from the Cascades region. Cores were dried at 35°C for two weeks in individual aluminum foil packets, then transferred to an air-tight plastic container with Drierite desiccant (Sigma-Aldrich, St. Louis, Missouri, USA). Our goal was to produce wood cores with a reduced moisture content comparable to kiln-dried lumber, but without exposing wood to the high temperatures used in kiln drying (90–100°C), to avoid driving off potentially diagnostic molecules. We also attempted to control the effect of wood age on subsequent chemical analyses by selecting identical growth years for analysis. The oldest growth year shared by all samples was 1986 (due to a small number of shallow cores in Coast Range trees), so our analyses in this study focused on years 1986, 1987, and 1988.

### Mass spectrometry

Mass spectra were acquired using an AccuTOF DART mass spectrometer (JEOL USA, Peabody, Massachusetts, USA) in positive ion mode. We used the DART source parameters as previously described for this particular instrument (Lancaster and Espinoza, 2012a, 2012b; Espinoza et al., 2014, 2015; McClure et al., 2015). We analyzed annual rings directly via DART-TOFMS by holding samples in the helium input stream for approximately eight seconds (McClure et al., 2015; Lesiak and Musah, 2016). We selected poly(ethylene glycol) 600 (Ultra Scientific, Kingstown, Rhode Island, USA) as our mass calibration standard, which we analyzed at the beginning and end of every set of samples from the same sampling location and after every third sample (McClure et al., 2015).

### Data analysis

**Fig. 2.**
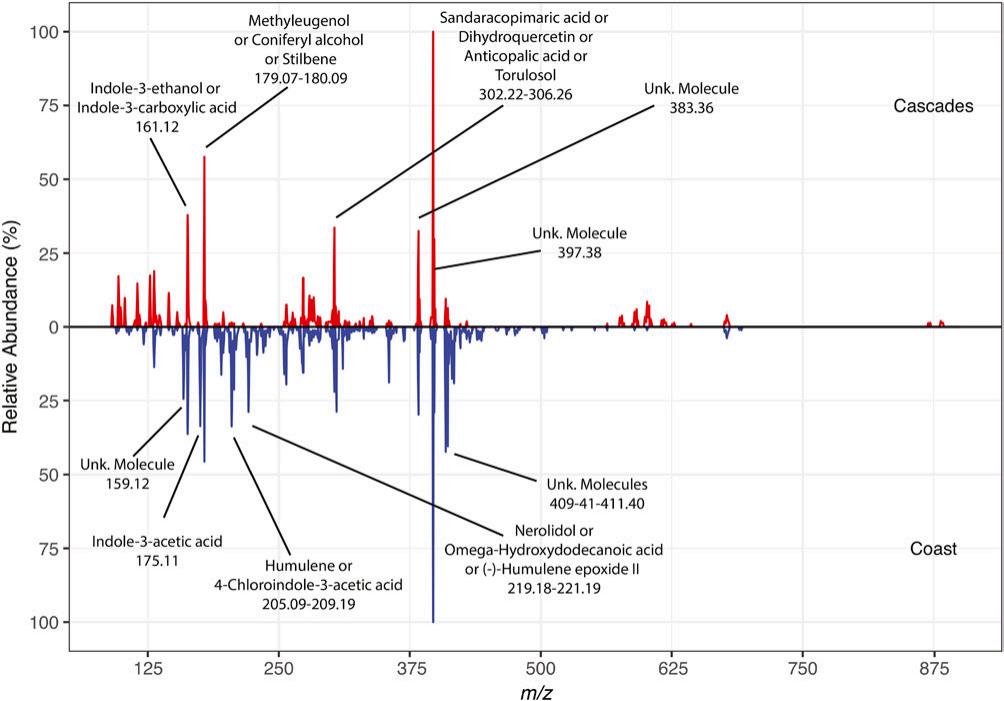
Graph of two aligned representative mass spectra. The *x*-axis shows the mass-to-charge ratio (*m/z*) and the *y*-axis shows molecule relative abundance (%). The red spectrum is a representative from the Cascades region (44.55878°N, 122.04321°W) and the blue spectrum is a representative from the Coast region reflected vertically (44.06787°N, 123.64871°W). We labeled molecule peaks with at least 25% relative abundance, some of which are unknown (Unk. Molecule). For peaks with similar *m/z*, we labeled a range of *m/z* with multiple names. Additionally, some molecules have multiple names for a single *m/z*, and we labeled all names that would fit in the limited space. Refer to Appendix S4 for the full list of molecules.

We analyzed our data using TSSPro3 (Shrader Analytical Laboratories, Detroit, Michigan, USA), Mass Mountaineer version 2 (RBC Software, Peabody, Massachusetts, USA), and R version 3.3.2 (R Core Team, 2016) with the packages randomForest version 4.6-12 (Liaw and Wiener, 2002), ROCR version 1.0-7 (Sing et al., 2005), vcfR version 1.3.0 (Knaus and Grunwald, 2016), ggplot2 version 2.2.0 (Wickham, 2009), and gridExtra version 2.2.1 (Auguie, 2016). We have provided relevant R code for the random forest analysis, including custom graphs (Appendix S1), as well as our raw data files (Appendices S2, S3). We used the TSSPro3 processing software to obtain mass spectra corresponding to: (1) each annual ring analyzed via DART-TOFMS (three mass spectra per individual; *n* = 560), and (2) a mass spectrum averaged over growth years 1986–1988 (one mass spectrum per individual; *n* = 188). Mass spectra include estimated mass-to-charge ratios (*m/z*) and relative molecule abundance (0–100%). Specifically, DART-TOFMS software outputs a mass spectrum in which each peak represents a different molecule, with its height normalized to that of the most abundant molecule. In this way, spectra are normalized within a spectrum, not globally across all spectra (Cody, 2015). The mass tolerance for the molecules detected in each mass spectrum was 250 mDa and the minimum relative abundance was 1%, which resulted in 946 potential molecules across all samples. Figure 2 shows two aligned representative mass spectra, one from the Cascade Range (Fig. 2 [red, 1987], 44.55878°N, 122.04321°W) and one from the Coast Range (Fig. 2 [blue, 1986], 44.06787°N, 123.64871°W). Using Mass Mountaineer, we were able to infer the identity of a subset of the most abundant molecules (Appendix S4; Shinbo et al., 2006).

To address our study questions, we used random forests classification from the R package randomForest to predict the class membership of each sample using mass spectra from DART-TOFMS. Random forest analysis is a classification method that is robust to nonnormal distributions (e.g., zero-truncated data, extreme value distributions) and can handle up to thousands of variables without the need for variable selection and without overfitting (Breiman, 2001; Strobl et al., 2009). We specified classification models to test four different grouping variables: Source for each individual annual ring, abbreviated Source*_INDIV_* (two classes: Cascades and Coast); Source for each tree averaged across annual rings, abbreviated Source*_MEAN_* (two classes: Cascades and Coast); Year (three classes: 1986, 1987, 1988); and Year*Source (six classes: Cascades 1986, Cascades 1987, Cascades 1988, Coast 1986, Coast 1987, Coast 1988). These models are summarized in Table 1.

**Table 1.** Abbreviations used to identify each classification model and a description of the grouping variable, classes within the grouping variable, and the number of samples used to train the model.

Model identifier	Grouping variable	Classes	*n*
Source*_INDIV_*	Region of origin	Cascades, Coast	560
Source*_MEAN_*	Region of origin	Cascades, Coast	188
Year	Growth year	1986, 1987, 1988	560
Year*Source	Growth year and region of origin	Cascades 1986, Cascades 1987, Cascades 1988, Coast 1986, Coast 1987, Coast 1988	560

*Note*: *n* = sample size.

Random forests were generated for each of our classification models considering all 946 molecules (classification variables) across sample mass spectra. We performed 500 iterations of the following protocol: (1) we randomly sampled an 80% subsample of mass spectra to be designated as the training set, from which a random forest of 500 classification trees was generated; (2) the median out-of-bag (OOB) classification error (Breiman, 2001, 2002; Liaw and Wiener, 2002) for the random forest was obtained; and (3) the remaining 20% subsample of mass spectra was designated a validation set to test the performance of the random forest for class membership prediction (Lever et al., 2016). Instability is a feature of random forest analysis, and complete reproducibility across replicate analyses cannot be assured (Breiman, 2001; Strobl et al., 2009). For this reason, we performed 500 iterations of each random forest model to better understand the distribution of classification values. Previous studies using DART-TOFMS for the classification of botanical samples have reported “classification accuracy” (Lancaster and Espinoza, 2012a, 2012b; Espinoza et al., 2014, 2015; McClure et al., 2015; Musah et al., 2015). To be consistent, we reported the complement of median OOB classification error, or “classification accuracy” (classification accuracy = 1 − classification error), so that our results could be directly compared to other DART-TOFMS studies. We measured overall classification accuracy and classification accuracy by class for the Source*_INDIV_* and Source*_MEAN_* models to test for classification asymmetry via a paired *t* test in R. To evaluate whether classification accuracy was higher than random expectations, we performed randomization tests (by shuffling class identifiers; 500 iterations) to determine the expected random accuracy for random forests.

For the Source*_INDIV_* and Source*_MEAN_* classification models, we used the R package ROCR to calculate the true positive and false positive rates of class prediction for the 20% validation set over 500 iterations (Gu et al., 2011; Xi et al., 2014). We displayed the performance of 500 random forests visually as receiver operating characteristic (ROC) curves, and used a generalized additive model and a cubic spline to generate a mean ROC curve over 500 iterations. Empirical measures of model performance are shown as the mean area under the ROC curve (AUC) for the 500 random forests.

To tentatively identify molecules, we compared mass-to-charge ratios from Douglas-fir spectra with a list of publicly available molecular masses from the conifer tree genera *Pseudotsuga* Carrière and *Pinus* L. using Mass Mountaineer (Shinbo et al., 2006). We also used the importance function of randomForest to obtain the Gini impurity index (Gini index) for the Source*_INDIV_* model and the Source*_MEAN_* model (Liaw and Wiener, 2002). Node impurity decreases each time a variable is used to partition data. After each partitioning event at a node, the samples remaining to be classified are more alike (i.e., belong to the same class) and descendent nodes have a lower node impurity. Variables that frequently partition data across random forests have a higher decrease in node impurity, which is estimated as a mean considering all 500 classification trees in the random forest (Breiman, 2001). The scale of the Gini index is based on the number of samples remaining to be classified after a variable is employed to partition samples (Breiman, 2001). A larger sample size to train the model, such as for the Source*_INDIV_* model, leads to a greater overall mean decrease in node impurity and Gini index. We compared lists of the 50 largest mean Gini indices from the Source*_INDIV_* model and the Source*_MEAN_* models to identify ions that were shared by both models (Venny version 2.1; Oliveros, 2007).

Finally, we generated a heat map of molecular masses and intensities for each averaged spectrum using the R package vcfR by applying a mass tolerance of 1 Da and a minimum relative abundance of 5%. Molecule relative abundance was log_2_ transformed to aid visualization of rare molecules. As described above, the abundance of each molecule is normalized row-wise (by sample), with 100% reflecting the most abundant molecule. Using available DART-TOFMS software (e.g., TSSPro3, Mass Mountaineer), total sample counts cannot be obtained, so normalization across samples cannot be made.

## RESULTS

### Classification

Our analysis evaluated the suitability of four classification models for Douglas-fir wood metabolites, including Source*_INDIV_*, Source*_MEAN_*, Year, and Year*Source (Table 1). The results from these analyses are summarized in Table 2 and described below.

**Table 2.** Results of the random forest classification analysis for each model.

		Estimated mean classification accuracy^a^	
Model	Class	Randomized (95% CI)	Observed (95% CI)
Source*_INDIV_*	2	49.8% (49.5, 49.3)	75.7% (75.6, 75.8)
Source*_MEAN_*	2	48.9% (48.5, 49.3)	70.1% (70.0, 70.2)
Year	3	32.9% (32.7, 33.1)	24.5% (24.4, 24.6)
Year*Source	6	16.2% (16.0, 16.3)	16.0% (15.9, 16.1)

a Estimated mean classification accuracies after 500 iterations for randomized and observed data; 95% confidence intervals are in parentheses. Estimated mean classification accuracy is the complement of the estimated mean of the median out-of-bag classification error for 500 iterations.

#### S*ource*_INDIV_ model

**Fig. 3.**
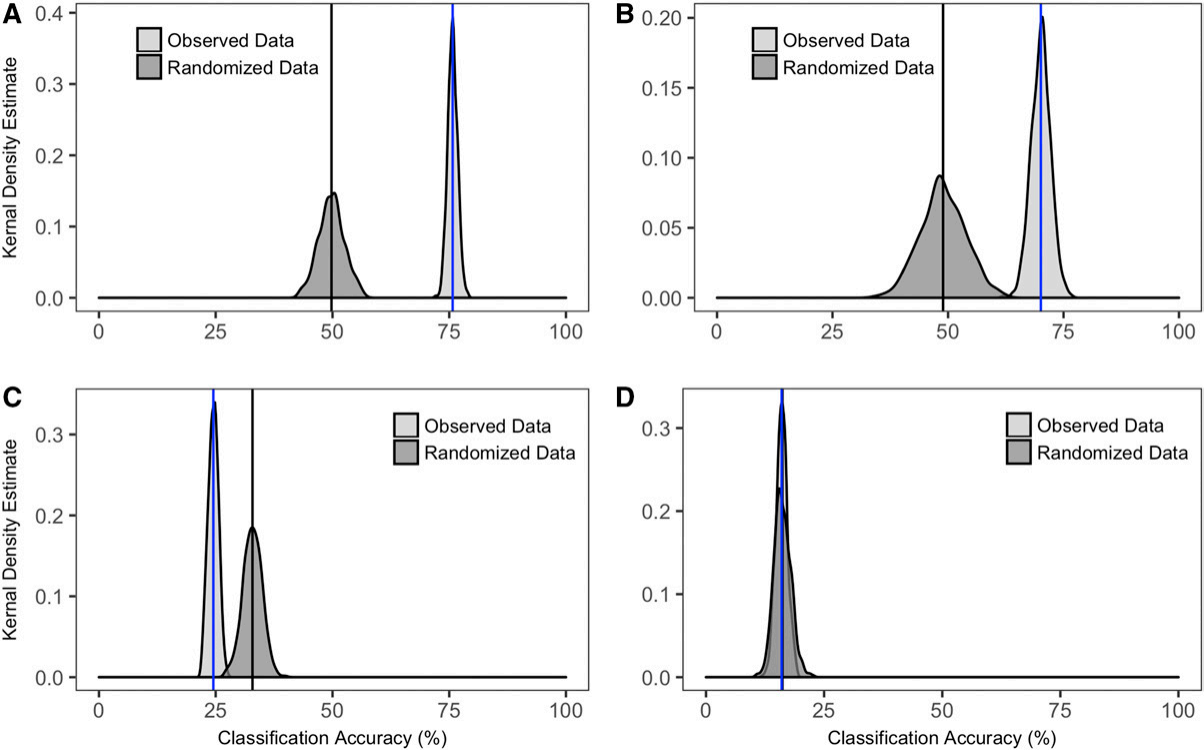
Distributions of the classification accuracies from random forests. Dark gray distributions were generated from randomized data, and light gray distributions were generated from observed data. Blue lines indicate the estimated mean classification accuracy for observed data, and black lines indicate the estimated mean classification accuracy for randomized data. 95% confidence intervals are listed in Table 2. Classification accuracies are shown for the Source*_INDIV_* (A), Source*_MEAN_* (B), Year (C), and Year*Source (D) models.

This random forest analysis was based on 500 classification trees across 500 iterations and tested classification accuracy arising from geographic source variation in wood chemistry. All individual annual rings were assigned to one of two location classes (Table 1). Our estimated mean classification accuracy of 75.7% for observed data is significantly higher than the estimated mean classification accuracy with randomized data (49.8%; Table 2, Fig. 3A).

#### S*ource*_MEAN_ model

This model also tested classification accuracy to geographic source variation in wood chemistry. Mean spectral abundance values for samples were assigned again to one of two location classes (Table 1). Random forest analysis based on 500 classification trees across 500 iterations returned an estimated mean classification accuracy of 70.1% for observed data, which is significantly higher than the estimated mean classification accuracy with randomized data (48.9%; Table 2, Fig. 3B).

#### Y*ear* model

This random forest analysis was based on 500 classification trees across 500 iterations to classify sample mass spectra by growth year (Table 1). Our random forest analysis with observed data returned an estimated mean classification accuracy of 24.5%. This value is significantly lower than the mean classification accuracy of 32.9% estimated from 500 randomizations (Table 2, Fig. 3C).

#### Year*S*ource* model

We used random forests to test the classification accuracy based on interannual and geographic source variation in wood chemistry. Samples were assigned one of six categories (Table 1). Our random forest analysis based on 500 classification trees across 500 iterations with observed data returned an estimated mean classification accuracy of 16.0%. The estimated mean classification accuracy from 500 randomizations was 16.2%, a value that is nearly identical to observed values (Table 2, Fig. 3D).

### Model performance

**Fig. 4.**
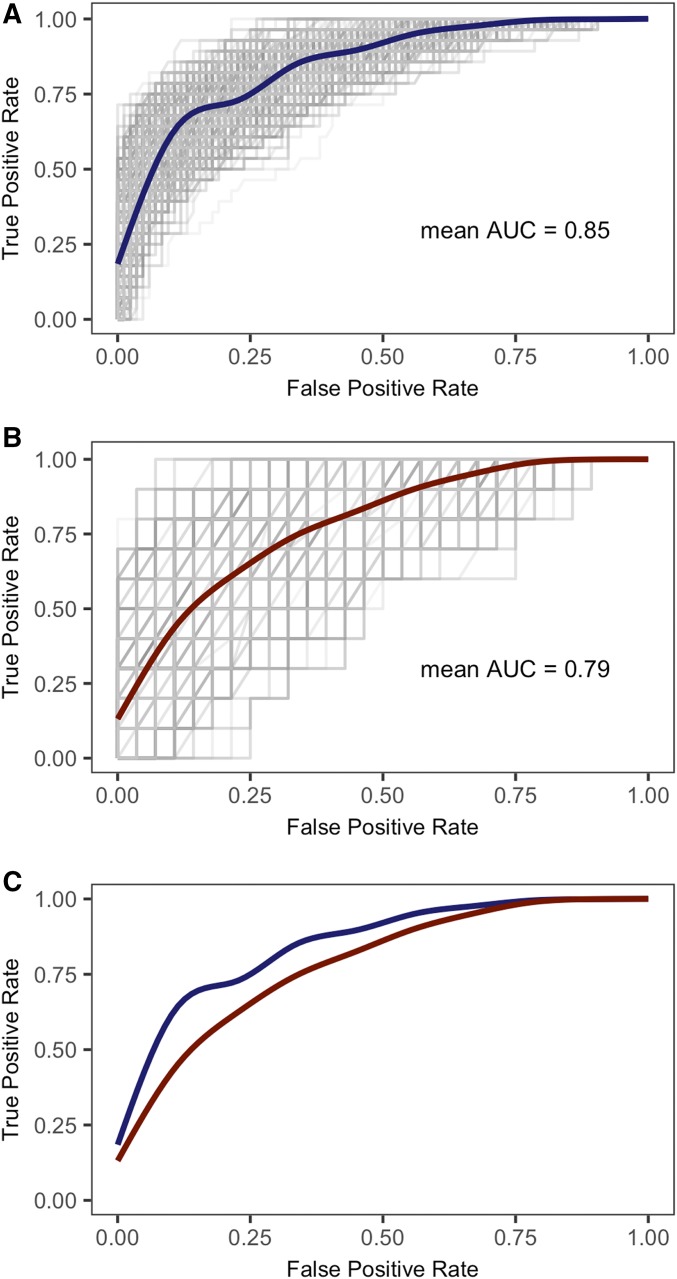
ROC curves generated for 500 random forests by predicting the class membership of each sample in a validation set. The *x*-axis is the false positive rate and the *y*-axis is the true positive rate. Gray lines indicate individual ROC curves from each of the 500 iterations. Colored lines indicate the estimated mean ROC curve generated with a generalized additive model and a cubic spline. (A) ROC plots for the Source*_INDIV_* model, (B) ROC plots for the Source*_MEAN_* model, and (C) superimposed mean ROC curves for the Source*_INDIV_* (blue) and the Source*_MEAN_* (red) models.

To assess model performance, we calculated the area under the ROC curve (AUC). The AUC of the Source*_INDIV_* model (0.85) was substantially higher than the Source*_MEAN_* model (0.79) (Fig. 4A, 4B), and direct comparison of mean model performance (Fig. 4C) showed that Source*_INDIV_* analysis performed better than the Source*_MEAN_* analysis. By conducting multiple iterations, we demonstrated that ROC curves (Fig. 4A, 4B; gray lines) are nonuniform across iterations and that the performance of each random forest and the AUC is dependent on samples included in the validation set.

### Molecule importance

**Fig. 5.**
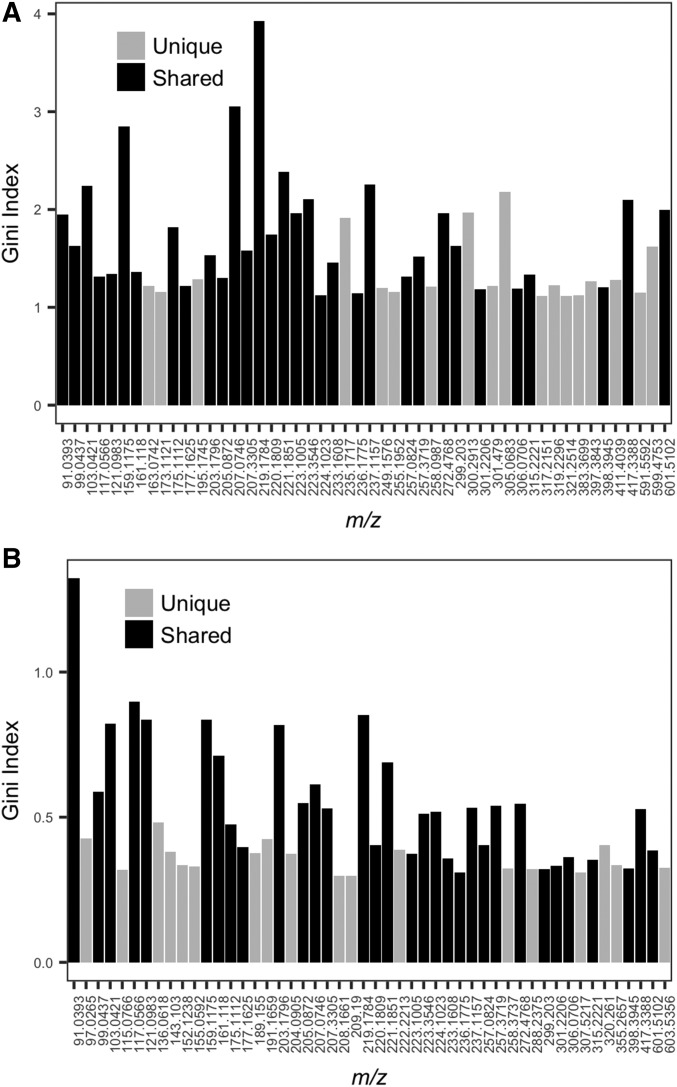
Comparison of the 50 molecules of highest Gini indices from the Source*_INDIV_* (A) and Source*_MEAN_* (B) models. Gray bars are unique to each model and black bars are molecules that are shared among the highest Gini indices for these models. The shared molecules were identified by comparing the highest Gini indices from both models using a Venn diagram with the program VENNY (Oliveros, 2007).

The 946 putative molecules detected from all samples showed a mass-to-charge range of 90.06 to 1060.90 *m/z*. Using Mass Mountaineer, we were able to infer the identity of 65 molecules (∼7%; Appendix S4; Shinbo et al., 2006). Well-known among characterized mass-to-charge ratios were molecules like the lignin precursor coniferyl alcohol (180.08 *m/z*; Quideau and Ralph, 1992), the methylated form of the plant auxin indole-3 acetic acid or methyl indole-3-acetate (189.08 *m/z*; Simon and Petrášek, 2011), the defense molecule pinosylvin (212.08 *m/z*; Jorgensen, 1961), the flavonolignan pseudotsuganol (236.18 *m/z*; Foo and Karchesy, 1989), and another conifer defense molecule, sandaracopimaric acid (302.22 *m/z*; Hall et al., 2013). By tabulating the 50 molecules with the highest Gini index for the Source*_INDIV_* model and the Source*_MEAN_* model (Fig. 5A, 5B, respectively), we found that 32 of the 50 highest Gini index molecules (64%) are shared among both models (Fig. 5A, 5B: black bars), and 18 are unique to each model. Of the 32 shared molecules, 14 (∼44%) were assigned a putative identity based on mass-to-charge ratio (Table 3).

**Table 3.** Putative identities for 14 of the 32 molecules that were shared among the lists of 50 molecules with the highest Gini indices from the Source*_INDIV_* and the Source*_MEAN_* models. Identities were approximated in Mass Mountaineer by comparing the mass-to-charge ratio of each molecule to a list of molecules identified in *Pinus* and *Pseudotsuga*. Provided are names that have been used to describe the molecules, their molecular formula, their mass-to-charge ratio, and the species from which they were identified.

Molecule name	Molecular formula	Mass (*m/z*)	Species
Indole-3-carboxylic acid	C_9_H_7_NO_2_	161.118	*Pinus banksiana*
Indole-3-ethanol	C_10_H_11_NO	161.118	*Pinus contorta*
Indole-3-acetic acid	C_10_H_9_NO_2_	175.11121	*Pinus contorta*, *P. grandis*
N6-(delta-2-isopentenyl)adenine	C_10_H_13_N_5_	203.1796	*Pinus halepensis*
(R)-(-)-alpha-curcumene	C_15_H_22_	203.1796	*Pinus halepensis*
(-)-Germacrene D, (-)-Isocaryophyllene, (-)-Zingiberene, (E)-beta-Bourbonene, (E)-Caryophyllene, (Z)-beta-Farnesene, alpha-Muurolene, beta-Gurjunene, beta-Sesquiphellandrene, Copaene, Cyclohexane, delta-Cadinene, gamma-Cadinene, gamma-Muurolene, Humulene, Longicyclene, longifolene	C_15_H_24_	205.0872	*Pinus armandii, P. cembra, P. contorta, P. eldarica, P. formosana, P. halepensis, P. kochiana, P. longifolia, P. sylvestris, Pseudotsuga menziesii, P. wilsoniana*
(-)-beta-caryophyllene epoxide, (-)-humulene epoxide II	C_15_H_24_O	221.1851	*Pinus longifolia*, *P. luchuensis*, *P. pallasiana*
(-)-alpha-cadinol, copaborneol, delta-cadinol, elemol, guaiol, nerolidol	C_15_H_26_O	223.10049	*Pinus pallasiana*, *P. palustris*, *P. parviflora*, *P. silvestris*, *P. sosnowskyi*
4-Chloroindole-3-acetic acid methyl ester	C_11_H_10_ClNO_2_	224.10229	*Pinus pallasiana*, *P. sylvestris*
ar-Pseudotsugonal	C_15_H_20_O_2_	233.1608	*Pinus sylvestris*
Atlantolone, pseudotsugonal	C_15_H_24_O_2_	237.11571	*Pinus sylvestris*
Pinocembrin	C_15_H_12_O_4_	257.0824	*Pinus sylvestris*, *P. taeda*
Abieta-7,13-diene	C_20_H_32_	272.47681	*Pinus thunbergi*, *Pseudotsuga wilsoniana*
6-C-Methylkaempferol	C_16_H_12_O_6_	301.22061	*Pseudotsuga wilsoniana*
Dehydroabietic acid	C_20_H_28_O_2_	301.22061	*Pseudotsuga japonica*, *P. wilsoniana*
(2R)-5,4′-Dihydroxy-7-methoxy-6-methylflavanone	C_17_H_16_O_5_	301.22061	*Pseudotsuga japonica*
Dehydroabietic acid	C_20_H_28_O_2_	301.22061	*Pseudotsuga menziesii*
13-Epitorreferol, 8-alpha,13S-epoxy-14-labden-6alpha-ol, torulosol	C_20_H_34_O_2_	306.07059	*Pseudotsuga menziesii*, *P. wilsoniana*
Catechin-4-beta-ol	C_15_H_14_O_7_	306.07059	*Pseudotsuga wilsoniana*
(2R,3R)-Pinobanksin 3-acetate, sylpin	C_17_H_14_O_6_	315.22211	*Pseudotsuga wilsoniana*

**Fig. 6.**
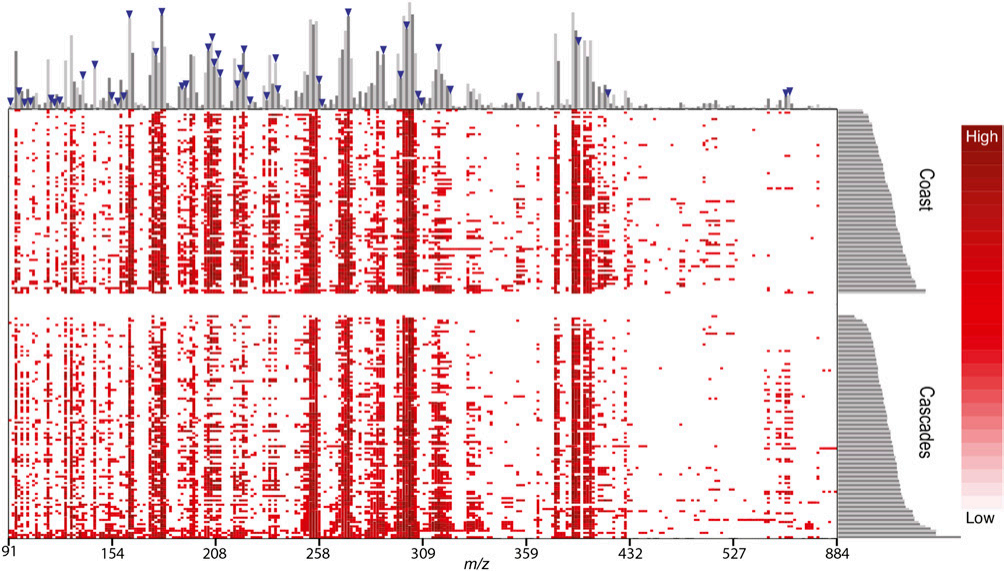
Heat map of wood samples showing the size distribution and relative abundance of wood-derived molecules. Rows indicate samples, and columns indicate molecule abundance, estimated as averaged mass spectra (Source*_MEAN_* model). Abundance is indicated by degree of red color (white = low abundance; red = high abundance), and blue triangles indicate molecules showing the approximate location of the 50 highest Gini indices from the Source*_MEAN_* model. Bar plots on the top and right axes indicate abundance sums, either by molecule (top) or individual sample (right).

A heat map of molecule abundances by sample from the Source*_MEAN_* model displays qualitative differences between samples originating from the Cascade and Coast ranges (Fig. 6). In this plot, molecules with the 50 highest Gini index values from the Source*_MEAN_* model are identified (Fig. 6: blue triangles). Both common and rare molecules have high Gini values, which is indicated by the bar plot of summed molecule abundances along the upper *x*-axis. Noteworthy differences between these populations can be observed in the 208–258 *m/z* range, where the Coast population has high abundances for many molecules; conversely, samples from the Cascade Range had higher abundances for many molecules in the 527–884 *m/z* range. Differences in these *m/z* ranges can also be seen in Fig. 2.

### Classification asymmetry

**Fig. 7.**
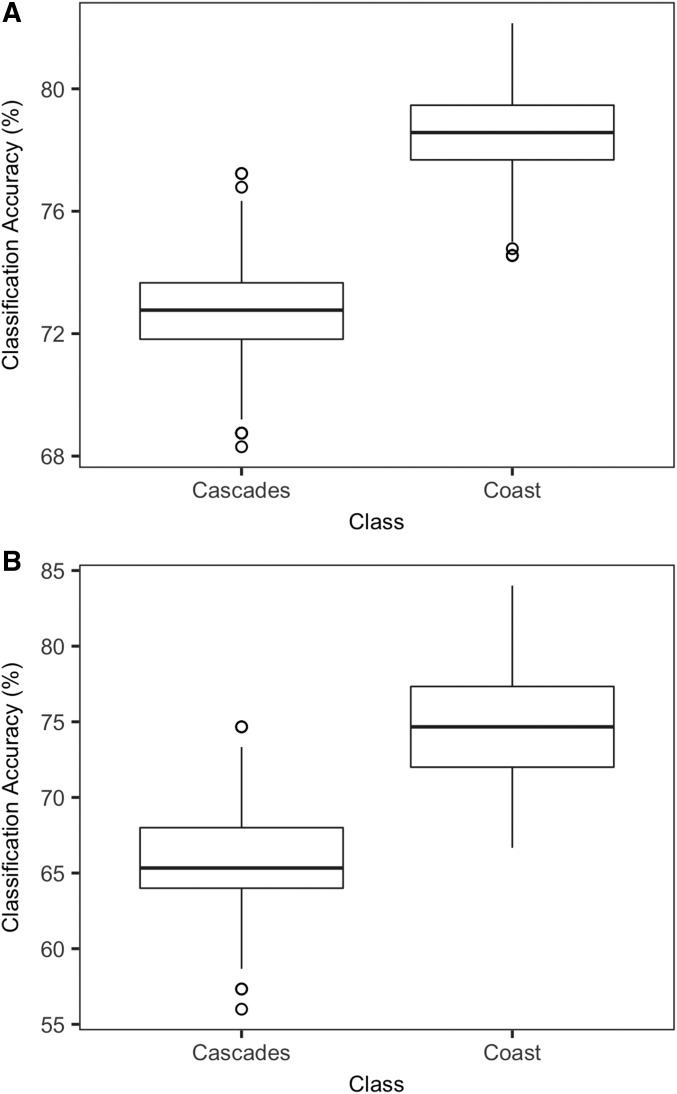
Box plots showing the difference in random forest classification accuracies for the Cascade Range class and Coast Range class based on 500 iterations of random forest analysis each with 500 classification trees. (A) Classification accuracies for Cascades and Coast classes based on 560 individual spectra (Source*_INDIV_* model). (B) Classification accuracies for Cascades and Coast classes based on 188 mean spectra (Source*_MEAN_* model).

Our comparison of the classification accuracy for the Cascades and Coast classes from the Source*_INDIV_* and Source*_MEAN_* models (Fig. 7A, 7B) revealed that across both analyses, classification accuracy was higher for the Coast trees (Source*_INDIV_* 78.5%, Source*_MEAN_* 74.6%) and lower for the Cascades trees (Source*_INDIV_* 72.7%, Source*_MEAN_* 65.5%), and the mean values were significantly different (Source*_INDIV_ t* = 59.915, df = 499, *P* < 0.001; Source*_MEAN_ t* = 48.632, df = 499, *P* < 0.001). This indicates that classification accuracy is nonidentical in reciprocal comparisons, and that in our specific case, classification accuracy of wood samples depends on the specific direction of the classification question.

## DISCUSSION

We addressed questions concerning the range of metabolite profile variation exhibited by Douglas-fir wood across geography and across years, and the accuracy of geographic classifications for individual trees based on DART-TOFMS spectra. Geographic classification models based solely on Source were the most accurate for both data treatments (Source*_INDIV_*, Source*_MEAN_*). Random forest mean classification accuracy was 75.7% for the Source*_INDIV_* model and 70.1% for the Source*_MEAN_* model. These values are significantly higher than random expectations (∼50%; Table 2; Fig. 3A, 3B). We attributed the higher classification accuracy in the Source*_INDIV_* model to the dependence between annual rings within individual Douglas-fir trees, as well as the larger sample size; simply decreasing the sample size for the Source*_INDIV_* model to 188 (same as the Source*_MEAN_* model) results in a decrease in classification accuracy (Appendix S5). Based on this analysis, we conclude that there is substantial geographic source differentiation between chemometric data derived from Douglas-fir tree cores separated by small geographic distances (e.g., Cascade and Coast ranges, ∼35–65 km) and that analyses based on multiple individual spectra (pseudoreplicates) perform as well as or better than those based on spectral means.

Moreover, we tested each random forest for the Source*_INDIV_* and Source*_MEAN_* data sets with a randomly selected validation set (unknowns) for 500 iterations. The Source*_INDIV_* models based on pseudoreplicates performed better when classifying unknowns than the Source*_MEAN_* models based on spectral means (Fig. 4C); again, the lower performance observed in the Source*_MEAN_* model was due to its smaller sample size (Appendix S5). The relationship between classification power/accuracy and reference sample size is relevant to forensic wood identification studies, as these analyses typically have a limited number of reference standards, regardless of the identification method used (genetic, isotopic, chemical, morphological). The small number of reference standards available for many CITES-protected tree species is due to the lack of diagnostic specimen vouchers (e.g., flowers, fruit, leaves) that can be used to convincingly identify wood samples to species, the limited availability of geographically source-identified wood specimens, and the ad hoc nature of adding reference materials derived from forensic investigations (Dormontt et al., 2015). The identification and acquisition of taxonomically validated, geographically referenced wood standards continues to be a principal focus for the wood forensics community.

The classification accuracy of individual spectra to Year classes (1986, 1987, or 1988) and Year*Source classes was virtually indistinguishable from random assignments (Table 2; Fig. 3C, 3D). These results suggested that chemometric variation across adjacent annual rings in Douglas-fir heartwood is indistinct, and that the variation is not adequately explained by year for samples collected over a wide geographic range. It is important to note that our samples capture chemometric variation from a small temporal (three consecutive years out of decades) and longitudinal (5 mm out of tens of meters) position from an adult Douglas-fir tree; more intensive sampling across the length and girth of a tree is required to fully understand intra-individual variation.

By ranking the 946 putative molecules detected via DART-TOFMS using the Gini index, we are able to identify the most important molecules for classifying mass spectra to geographic origin for the Source*_INDIV_* and Source*_MEAN_* models. The complete analysis based on 946 putative molecules is effectively a “first-pass” analysis for screening variable importance; it is possible to use this analysis to select more-informative subsets of molecules for subsequent analysis. For example, reducing the full list of 946 predictor variables down to the 50 variables with the highest-ranking molecules according to the Gini index improves our classification accuracy from 75.7% to 76.8% for the Source*_INDIV_* model and from 70.1% to 74.1% for the Source*_MEAN_* model (Appendix S6).

An important observation of our classification experiment was that misclassification (false positives and false negatives) for the Cascades and Coast classes are asymmetrical, with misclassifications more frequent in Cascades-derived wood samples than Coast-derived wood samples for both source models (Source*_INDIV_* and Source*_MEAN_*; Fig. 7A, 7B). This observation suggests that for illegal logging studies, the classification power of a specific question may depend on the direction of classification. For example, in a hypothetical scenario of “classifying stolen Douglas-fir wood,” the distribution of classification accuracy makes it easier to correctly classify unknown trees to their geographic source if they derived from the Coast Range than if they had derived from the Cascade Range.

Finally, while we were able to measure geographic differences in Douglas-fir wood chemistry using DART-TOFMS, we were not able to identify whether this variation is a consequence of climatic, edaphic, or genetic factors, individually or combined. Chemical analysis of wood samples from provenance and reciprocal transplant tests (Gould et al., 2012) could shed light on the contribution of these factors to variation in wood chemistry. For example, in a recently established “Seed Source Movement Trial” (Gould et al., 2012; Ford et al., 2016), 60 half-sib families of Douglas-fir have been planted at nine sites across the Pacific Northwest, spanning a range of climates from coastal to montane. Cores from these populations would show the contribution of genetic background and growth environment to DART-TOFMS profiles. These types of studies are a logical next step for understanding spatial variations in DART-TOFMS data derived from wood.

### Classification methods for DART-TOFMS data

Interpretation of sample classifications based on mass spectrometry–derived data have relied on a number of approaches, including principal component analysis (Pan et al., 2007; Musah et al., 2015), linear and kernel discriminant analysis (Lancaster and Espinoza, 2012a, 2012b; Espinoza et al., 2014, 2015; McClure et al., 2015), partial least square-discriminant analysis (Gu et al., 2011; Lee et al., 2012; Kim et al., 2015), support vector machines (Mahadevan et al., 2008; Zhou et al., 2010), and random forest (Baniasadi et al., 2013). Previous studies using DART-TOFMS, in particular for wood identification, have primarily used linear and kernel discriminant analysis.

To provide a comparison to other methods, we also analyzed our Douglas-fir wood mass spectra with linear discriminant analysis (LDA) and calculated classification accuracy using leave-one-out cross-validation (Appendix S7). For our data, the difference between LDA and random forest classification methods was minimal, as the LDA-based classification accuracies were 72.9% for the Source*_INDIV_* and Source*_MEAN_* models (Appendix S7: Table S7.1). Despite the equivalence of random forests and LDA classification models in our example, random forests classification offers two significant advantages for DART-TOFMS data analysis. First, classification variables need to be selected a priori for LDA; in DART-TOFMS data, this is accomplished by choosing a “representative” spectrum from the pool of samples and evaluating compounds present in the representative spectrum. This step has the potential to bias the analysis (overfitting to the reference spectrum) and ignore less frequent, spatially important compounds. By contrast, random forest evaluates all classification variables, and it ranks their importance to the classification model. Second, random forest can include any kind of classification variable (categorical, ordinal, continuous, ratio) from any distribution. This makes it a potentially ideal method for incorporating and evaluating multiple sources of information (e.g., DART-TOFMS, genetic, and anatomic) in direct combined analyses.

### Other applications for DART-TOFMS analysis of wood

In addition to the promise of wood identification by DART-TOFMS metabolite profiling, the rapidity and ease of DART-TOFMS analysis make it a promising tool for addressing chemometric questions in other disciplines. In our study, we estimated the identity of a mere 65 (∼7%) of the 946 putative molecules detected by DART-TOFMS (Appendix S4). That is, the majority of the molecules detected in Douglas-fir wood have yet to be identified and/or included in mass spectrometry databases (Shinbo et al., 2006). Identifying the complete spectrum of molecules in wood is a critical first step to understanding the role that these molecules play in economically important wood quality traits such as strength, elasticity, and fitness traits like resistance to burrowing insects and wood rot fungi.

Considerable attention has been given to annual rings as environmental records of climate change (Fritts, 1972; Crowley, 2000; Belmecheri et al., 2016). Given its sensitivity and small sample requirements (∼20 mm^3^ for this study), DART-TOFMS analysis of annual rings could be conducted over centuries of growth from different populations and species, and this offers a method to study intra-individual and population-level plant chemical responses across geography and time. Although we have demonstrated that growth year is a poor predictor of chemical variation, the relationship between wood chemistry and climate over longer periods of time (decades to centuries) is unexplored. Particularly interesting questions for the response of Douglas-fir to climatic variation are induced elevated terpene synthase activity with exposure to high temperatures (Litvak et al., 2002) and the suppression of wound response after light and water stress in conifers (McGarvey and Croteau, 1995). By combining historical weather records and historical metabolite profiles, it should be possible to identify climatically responsive molecules present in wood, and use these to make predictions about how wood composition will change with different models of predicted future climate warming (McIntyre et al., 2015).

### Conclusions

Rapid screening methods for identifying the species and geographic provenance of commercially traded wood are essential for enforcing illegal logging provisions outlined in the U.S. Lacey Act of 2008, the European Union Timber Regulation of 2010, and CITES. Numerous methodological approaches are currently being evaluated and applied, including DNA genotyping, stable isotope composition analysis, and wood chemometric analysis (Dormontt et al., 2015). Studies have demonstrated that DART-TOFMS is one of the most rapid screening tools available (Cody et al., 2005; Cody, 2013; Musah et al., 2015) and that it can differentiate molecules present in wood that show fixed or nearly fixed differences between tree species (Cody et al., 2012; Lancaster and Espinoza, 2012a, 2012b; Espinoza et al., 2014, 2015). Our study highlights the potential for using DART-TOFMS to identify the geographic origin of wood at scales under 100 km. In total, these studies show that DART-TOFMS can be used to address wood differences and wood identification at many scales—between populations, species, and genera.

## Supplementary Material

Supplementary Material 1

Supplementary Material 2

Supplementary Material 3

Supplementary Material 4

Supplementary Material 5

Supplementary Material 6

Supplementary Material 7

## Appendix

**Appendix 1.** GPS coordinates of Douglas-fir sampling locations, elevation, a priori source classifications, and number of trees sampled.

Population ID	Latitude (DD)	Longitude (DD)	Elevation (ft.)	Source	*n*
1024	44.30486	−122.84895	1393	Cascade Range	4
1026	44.414	−122.672	556	Cascade Range	2
1191	44.61435	−123.53946	696	Coast Range	4
1195	44.33089	−123.86224	1009	Coast Range	4
1202	44.60163	−121.95015	2615	Cascade Range	4
1223	44.19244	−121.98228	3354	Cascade Range	4
2034	43.21882	−122.1983	5278	Cascade Range	4
2092	44.36678	−122.02457	3467	Cascade Range	4
3031	44.77376	−122.54893	1089	Cascade Range	4
3054	44.1584	−122.62379	1548	Cascade Range	4
3061	44.17607	−122.99362	1942	Cascade Range	6
3175	44.18	−123.444	751	Coast Range	4
3187	43.33717	−123.55252	2127	Coast Range	3
3198	44.06787	−123.64871	2124	Coast Range	4
3202	45.33647	−123.65208	1837	Coast Range	4
3205	43.06919	−124.0074	1191	Coast Range	4
3218	43.31859	−124.07347	292	Coast Range	4
3238	43.82875	−123.35144	694	Coast Range	4
3240	44.2391	−123.436756	1623	Coast Range	3
3313	43.7085	−123.50705	692	Coast Range	4
3353	44.95717	−123.80177	2353	Coast Range	4
3358	44.38269	−123.46298	1303	Coast Range	4
3364	44.18041	−123.6151	1879	Coast Range	3
4005	44.435	−121.715	3311	Cascade Range	4
4069	45.56364	−121.51936	2420	Cascade Range	4
4085	45.28366	−121.68187	4160	Cascade Range	4
4126	43.3052	−122.78975	2651	Cascade Range	4
4146	43.63619	−122.42519	1666	Cascade Range	4
4153	43.52632	−122.43086	2948	Cascade Range	4
4158	43.74414	−122.54805	2102	Cascade Range	4
4173	44.19257	−122.30781	1963	Cascade Range	2
4192	44.366	−122.237	2783	Cascade Range	4
4193	44.39371	−122.2438	1676	Cascade Range	4
4194	44.373	−122.38	2150	Cascade Range	4
4196	44.433	−122.425	2371	Cascade Range	4
4199	44.418	−122.379	2389	Cascade Range	4
4202	44.66695	−122.11407	2725	Cascade Range	4
4203	44.79057	−122.0533	2573	Cascade Range	5
4205	44.432	−122.002	3331	Cascade Range	4
4209	44.55878	−122.04321	3869	Cascade Range	4
6015	45.318502	−123.85525	1376	Coast Range	4
6024	44.15252	−123.7439	720	Coast Range	4
6090	44.88084	−123.8743	1220	Coast Range	4
6095	44.11648	−124.07047	900	Coast Range	4
6105	44.52202	−123.76382	1543	Coast Range	4
6107	44.11923	−124.02402	1843	Coast Range	4
6118	43.731	−123.952	975	Coast Range	4
AMY	44.19366	−123.50253	739	Coast Range	4

*Note*: DD = decimal degrees; *n* = sample size.
