# Supplementary material for: Source identification of western Oregon Douglas-fir wood cores using mass spectrometry and random forest classification
Source: Appl Plant Sci. 2017 May 12;5(5):apps.1600158. doi: 10.3732/apps.1600158 (PMC5435404; doi:10.3732/apps.1600158)
Supplement: Supplementary file 1 [file apps.1600158_s1.pdf]

Appendix S1.

R Markdown document presenting sample R-code.
Prepared by: Kristen Noelle Finch, 24 February 2017
Code contributor: Javier Tabima

```
#Load packages
library(randomForest)
library(ggplot2)
library(dplyr)
library(ROCR)
library(gridExtra)
```

Recommended

Set your working directory with: setwd() or use the "Session" Menu > "Set working directory" > "Choose Directory"

Below is sample code that I ran to generate these data. This runs for 500 iterations, so the data used for graphed results have been saved independently and loaded subsequently.

Table S1.1. Explanation of abbreviations that appear in code.

Abbreviation	Model
v.r.2	SourceMEAN
a.r.2	SourceINDIV
a.s.3	Year
a.r.s.6	Year*Source

SourceMEAN Observed Data

Subset your data into a training and validation set. BUT MAKE SURE THAT THE CLASSES ARE BALANCED! I have 188 samples for the SourceMEAN model. 80% of 188 is ~150 and 150/2 is 75. There are 85 Coast samples and Coast is at a deficit, so this will work. 75 Coast, 75 Cascades, and the remaining samples will be reserved for the validation set (38).

```
# v.r.2 observed
df_250_1 <- read.csv("Cascade vs Coast 250 mmu and 1.csv")
# subset to keep variables for averaging
df_250_1 <- df_250_1[c(4:952)]
# dplyr functions
avg <- df_250_1 %>% group_by(Region, Pop, Ind)
indavg <- avg %>% summarise_each(funs(mean))
write.csv(indavg, "source_mean_data.csv")

# now we just want the grouping variable we are
```

```
# interested in an molecular abundance variables.
df <- indavg[c(1, 4:949)]
# You need a dataset that contains your predictor
# variables (ions) and one column that contains the
# class information.

# For Loop to run random forest X times. We create
# an empty list, to fill using the values of the
# fit and other elements

fit.list <- list()
roc.list <- list()
pred.list <- list()
for (i in 1:length(c(1:500))) {
    # for all items (i) in the list of chosen
    # length--change to number of iterations you want
    # to perform.
    cat("Repetition number ")  #Repetition number X will let us know that our loop is working.
    cat(i)
    cat("\n")

    # randomly select 75 from each; saved as a list
    cas.80 <- sample(nrow(cas), 75)
    coa.80 <- sample(nrow(coa), 75)
    # make new dataframe that matches the samples in
    # the list cas.80. This will become the Cascades
    # portion of the dataset to train the Random Forest
    # analysis.
    df.train.cas <- cas[cas.80, ]
    df.validate.cas <- cas[-cas.80, ]
    # make new dataframe that DOES NOT match the
    # samples in the list cas.80, and repeat the same
    # protocol to make dataframes for the Coast
    # portions of the training and validation tests.
    df.train.coa <- coa[coa.80, ]
    df.validate.coa <- coa[-coa.80, ]
    # Combine the Cascades and Coast portions of the
    # training and validation sets.
    df.train <- rbind(df.train.cas, df.train.coa)
    df.validate <- rbind(df.validate.cas, df.validate.coa)

    # this will run randomForest for each (i) in
    # fit.list 500 times.
    fit.list[[i]] <- (fit.forest <- randomForest(Region ~
        ., data = df.train, importance = TRUE))  #Grows the forest

    # this next part uses the model we built to
    # classify the validation set.
    forest.pr <- predict(fit.forest, type = "prob",
        df.validate)[, 2]
    forest.pred <- prediction(forest.pr, df.validate$Region)
    # We want to save the True Positive rate and the
    # False positive rate. These will then become the x
```

```
    # and y values for the ROC curve.
    forest.pref <- performance(forest.pred, "tpr",
        "fpr")
    pred.list[[i]] <- forest.pred
    # Saving x and y values in a dataframe
    df.forest <- data.frame(unlist(forest.pref@x.values),
        unlist(forest.pref@y.values))
    df.forest$replicate <- rep(i, nrow(df.forest))
    colnames(df.forest) <- c("x_axis", "y_axis", "replicate")
    roc.list[[i]] <- df.forest  #save the data frame as a list.
}  #end of for loop

# To see the distributions of the median of OOB
# error, you can use the list you just created and
# do a simple for loop again: calculate the median
# OOB classification error for each interation of
# the randomForest.
fit.median <- list()
for (i in 1:length(fit.list)) {
    fit.median[[i]] <- median(fit.list[[i]]$err.rate[,
        "OOB"])
}
# Unlisting the values err.obs.v.r.2
fit.median <- unlist(fit.median)
err.obs.v.r.2 <- data.frame(fit.median)  #write this out write.csv() >>>>

# use these values to make a density curve of the
# median OOB classification errors for 500
# iterations.

# ROC: Binding and creating the curve
roc.total.obs.v.r.2 <- as.data.frame(do.call(rbind,
    roc.list))
# write this out write.csv() >>>>

# Calculate area under the curve for each curve
auc.list <- list()
for (i in 1:length(pred.list)) {
    auc <- performance(pred.list[[i]], "auc")
    auc.list[[i]] <- unlist(slot(auc, "y.values"))
}

# stats to report.
auc.v.r.2 <- unlist(auc.list)
meanauc.obs.v.r.2 <- mean(round(auc, digits = 2))
auc.error.obs.v.r.2 <- qt(0.975, df = length(auc.v.r.2) -
    1) * sd(auc.v.r.2)/sqrt(length(auc.v.r.2))
auc.left.obs.v.r.2 <- meanauc.obs.v.r.2 - auc.error.obs.v.r.2
auc.right.obs.v.r.2 <- meanauc.obs.v.r.2 + aucerror.obs.v.r.2
auc.v.r.2.stats <- data.frame(cbind(meanauc.obs.v.r.2,
    auc.error.obs.v.r.2, auc.left.obs.v.r.2, auc.right.obs.v.r.2))
names(auc.v.r.2.stats) <- c("mean", "sd", "lower",
    "upper")
```

```
auc.v.r.2.stats$model <- c("v.r.2")
# write this out write.csv() >>>>
```

Now do the same with randomized data, but this time we are not interested in the ROC curve.

SourceMEAN Randomized Data

```
# v.r.2 randomized make your empty lists
fit.list <- list()

# start your for loop in the same way for all items
# (i) in the list of chosen length--change to
# number of iterations you want to perform.
for (i in 1:length(c(1:500))) {
    cat("Repetition number ")  #Repetition number X will let us know that our loop is working.
    cat(i)
    cat("\n")
    # reload the dataset.
    df <- indavg[c(1, 4:949)]
    # make a new dataframe containing the grouping
    # variable.
    rand.col.1 <- data.frame(df$Region)
    # delete the grouping variable in the main
    # dataframe.
    df$Region <- NULL
    # shuffle the values in the grouping variable
    rand.col.1 <- data.frame(rand.col.1[sample(nrow(rand.col.1)),
        ])
    # rename the grouping variable
    names(rand.col.1)[1] <- "Region"
    # recombine the main dataframe with the randomized
    # values in the grouping variable. Now the data
    # have been maintained, but the classes of the
    # grouping variable have been randomized.
    df <- cbind(rand.col.1, df)
    # Now we have to subset the dataset to obtain the
    # 80% training set and the 20% validation set,
    # taking care to maintain balanced class sample
    # sizes.
    cas <- subset(df, Region == "Cascades")
    coa <- subset(df, Region == "Coast")
    cas.80 <- sample(nrow(cas), 75)
    coa.80 <- sample(nrow(coa), 75)

    df.train.cas <- cas[cas.80, ]
    df.validate.cas <- cas[-cas.80, ]
    df.train.coa <- coa[coa.80, ]
    df.validate.coa <- coa[-coa.80, ]

    df.train <- rbind(df.train.cas, df.train.coa)
    df.validate <- rbind(df.validate.cas, df.validate.coa)

    # randomForest for 500 iterations
    fit.list[[i]] <- (fit.forest <- randomForest(Region ~
```

```
        ., data = df.train, importance = TRUE))  #Grows the forest
}  #end of for loop

# Obtain OOB error rates.
fit.median <- list()
for (i in 1:length(fit.list)) {
    fit.median[[i]] <- median(fit.list[[i]]$err.rate[,
        "OOB"])
}
# Unlisting the values to make a dataframe of OOB
# values for Randomized data.  err.rand.v.r.2
fit.median <- unlist(fit.median)
err.rand.v.r.2 <- data.frame(fit.median)  #write this out write.csv() >>>>
```

SourceINDIV Observed Data

Subset your data into a training and validation set. BUT MAKE SURE THAT THE CLASSES ARE BALANCED! For my data this required some calculations. I have 560 samples for the SourceINDIV model. 80% of 560 is 448 and 448/2 is 224. There are 252 samples for the Coast class which is at a deficit, and 224 will work. 224 Coast samples, 224 Cascades samples, and the remaining samples will be used for the validation set (112).

```
# a.r.2 observed
df_250_1 <- read.csv("Cascade vs Coast 250 mmu and 1.csv")
df <- df_250_1[c(4, 7:952)]

fit.list <- list()
roc.list <- list()
pred.list <- list()
for (i in 1:length(c(1:500))) {
    # for all items (i) in the list of chosen
    # length--change to number of iterations you want
    # to perform.
    cat("Repetition number ")  #Repetition number X will let us know that our loop is working.
    cat(i)
    cat("\n")

    cas <- subset(df, Region == "Cascades")
    coa <- subset(df, Region == "Coast")
    cas.80 <- sample(nrow(cas), 224)
    coa.80 <- sample(nrow(coa), 224)

    df.train.cas <- cas[cas.80, ]
    df.validate.cas <- cas[-cas.80, ]
    df.train.coa <- coa[coa.80, ]
    df.validate.coa <- coa[-coa.80, ]
    df.train <- rbind(df.train.cas, df.train.coa)
    df.validate <- rbind(df.validate.cas, df.validate.coa)

    # randomForest
    fit.list[[i]] <- (fit.forest <- randomForest(Region ~
        ., data = df.train, importance = TRUE))  #Grows the forest

    # this next part uses the model we built to
```

```
    # classify the validation set.
    forest.pr <- predict(fit.forest, type = "prob",
        df.validate)[, 2]
    forest.pred <- prediction(forest.pr, df.validate$Region)
    forest.pref <- performance(forest.pred, "tpr",
        "fpr")
    pred.list[[i]] <- forest.pred
    df.forest <- data.frame(unlist(forest.pref@x.values),
        unlist(forest.pref@y.values))
    df.forest$replicate <- rep(i, nrow(df.forest))
    colnames(df.forest) <- c("x_axis", "y_axis", "replicate")
    roc.list[[i]] <- df.forest  #save the data frame as a list.
}  #end of for loop

# Build dataframe of median of OOB error
fit.median <- list()
for (i in 1:length(fit.list)) {
    fit.median[[i]] <- median(fit.list[[i]]$err.rate[,
        "OOB"])
}
# Unlisting values and save err.obs.a.r.2
fit.median <- unlist(fit.median)
err.obs.a.r.2 <- data.frame(fit.median)
# write this out write.csv() >>>>

# ROC: Binding and creating the curve
roc.total.obs.a.r.2 <- as.data.frame(do.call(rbind,
    roc.list))
# write this out write.csv() >>>>

# Calculate area under the curve for each curve
auc.list <- list()
for (i in 1:length(pred.list)) {
    auc <- performance(pred.list[[i]], "auc")
    auc.list[[i]] <- unlist(slot(auc, "y.values"))
}

# stats to report.
auc.a.r.2 <- unlist(auc.list)
meanauc.obs.a.r.2 <- mean(round(auc.a.r.2, digits = 2))
auc.error.obs <- qt(0.975, df = length(auc.a.r.2) -
    1) * sd(auc.a.r.2)/sqrt(length(auc.a.r.2))
auc.left.obs <- meanauc.obs.a.r.2 - auc.error.obs.a.r.2
auc.right.obs <- meanauc.obs.a.r.2 + aucerror.obs.a.r.2
auc.a.r.2.stats <- data.frame(cbind(meanauc.obs.a.r.2,
    auc.error.obs.a.r.2, auc.left.obs.a.r.2, auc.right.obs.a.r.2))
names(auc.a.r.2.stats) <- c("mean", "sd", "lower",
    "upper")
auc.a.r.2.stats$model <- c("a.r.2")
# write this out write.csv() >>>>
```

SourceINDIV Randomized Data

```
# a.r.2 randomized
fit.list <- list()
for (i in 1:length(c(1:500))) {
    # for all items (i) in the list of chosen
    # length--change to number of iterations you want
    # to perform.
    cat("Repetition number ")  #Repetition number X will let us know that our loop is working.
    cat(i)
    cat("\n")
    # reload dataset
    df_250_1 <- read.csv("Cascade vs Coast 250 mmu and 1.csv")
    df <- df_250_1[c(4, 7:952)]
    # make a new dataset with the grouping variable
    rand.col.1 <- data.frame(df$Region)
    # remove the grouping variable from the main
    # dataset
    df$Region <- NULL
    # randomize the values in the grouping variable
    rand.col.1 <- data.frame(rand.col.1[sample(nrow(rand.col.1)),
        ])
    names(rand.col.1)[1] <- "Region"
    # recombine the grouping variable and the rest of
    # the molecular abundance data.
    df <- cbind(rand.col.1, df)

    # now subset the data into a training and
    # validation set.
    cas <- subset(df, Region == "Cascades")
    coa <- subset(df, Region == "Coast")
    cas.80 <- sample(nrow(cas), 224)
    coa.80 <- sample(nrow(coa), 224)

    df.train.cas <- cas[cas.80, ]
    df.validate.cas <- cas[-cas.80, ]
    df.train.coa <- coa[coa.80, ]
    df.validate.coa <- coa[-coa.80, ]
    df.train <- rbind(df.train.cas, df.train.coa)
    df.validate <- rbind(df.validate.cas, df.validate.coa)

    # Run randomForest
    fit.list[[i]] <- (fit.forest <- randomForest(Region ~
        ., data = df.train, importance = TRUE))  #Grows the forest
}  #end of for loop

# obtain OOB's and calculate medians.
fit.median <- list()
for (i in 1:length(fit.list)) {
    fit.median[[i]] <- median(fit.list[[i]]$err.rate[,
        "OOB"])
}
# Unlisting the values err.rand.a.r.2
fit.median <- unlist(fit.median)
err.rand.a.r.2 <- data.frame(fit.median)  #write this out write.csv() >>>>
```

```
# save data from the SourceMEAN and SourceINDIV
# model analyses
err.a.r.2 <- cbind(err.obs.a.r.2, err.rand.a.r.2)
names(err.a.r.2)[1] <- "Observed"
names(err.a.r.2)[2] <- "Randomized"
write.csv(err.a.r.2, "err.a.r.2.csv")
dens.a.r.2 <- err.a.r.2
temp <- err.a.r.2
temp[1] <- NULL
names(temp)[1] <- "OOB.med.err"
temp$group <- rep("Randomized", length(temp))
dens.a.r.2[2] <- NULL
names(dens.a.r.2)[1] <- "OOB.med.err"
dens.a.r.2$group <- rep("Observed", length(dens.a.r.2))
dens.a.r.2 <- rbind(dens.a.r.2, temp)
write.csv(dens.a.r.2, "dens.a.r.2.csv")

err.v.r.2 <- cbind(err.obs.v.r.2, err.rand.v.r.2)
names(err.v.r.2)[1] <- "Observed"
names(err.v.r.2)[2] <- "Randomized"
write.csv(err.v.r.2, "err.v.r.2.csv")
dens.v.r.2 <- err.v.r.2
temp <- err.v.r.2
temp[1] <- NULL
names(temp)[1] <- "OOB.med.err"
temp$group <- rep("Randomized", length(temp))
dens.v.r.2[2] <- NULL
names(dens.v.r.2)[1] <- "OOB.med.err"
dens.v.r.2$group <- rep("Observed", length(dens.v.r.2))
dens.v.r.2 <- rbind(dens.v.r.2, temp)
write.csv(dens.v.r.2, "dens.v.r.2.csv")

auc.stats <- data.frame(rbind(auc.v.r.2.stats, auc.a.r.2.stats))
write.csv(auc.stats, "auc.stats.csv", row.names = FALSE)
```

Year Observed Data

Subset your data into a training and validation set. BUT MAKE SURE THAT THE CLASSES ARE BALANCED! For my data this required some calculations. The smallest class (1986) in the grouping variable had 185 samples. 185 was set as the upper limit for each class. For this analysis, we are not interested in validating unknowns.

```
# a.s.3 observed
fit.list <- list()
for (i in 1:length(c(1:500))) {
    # for all items (i) in the list of chosen
    # length--change to number of iterations you want
    # to perform.
    cat("Repetition number ")  #Repetition number X will let us know that our loop is working.
    cat(i)
    cat("\n")

    df_250_1 <- read.csv("Cascade vs Coast 250 mmu and 1.csv")
    df_250_1$Year <- factor(df_250_1$Year)
```

```
    df <- df_250_1[c(2, 7:952)]
    is.factor(df$Year)  #this must be TRUE.

    # Divide the dataset into balanced classes of 185
    # samples each.
    year.86 <- subset(df, Year == "86")
    year.87 <- subset(df, Year == "87")
    year.88 <- subset(df, Year == "88")
    year.86 <- year.86[sample((1:nrow(year.86)), 185,
        replace = FALSE), ]
    year.87 <- year.87[sample((1:nrow(year.87)), 185,
        replace = FALSE), ]
    year.88 <- year.88[sample((1:nrow(year.88)), 185,
        replace = FALSE), ]

    df.train <- rbind(year.86, year.87, year.88)

    # run randomForest
    fit.list[[i]] <- (fit.forest <- randomForest(Year ~
        ., data = df.train, importance = TRUE))  #Grows the forest

}  #end of for loop

# Get median OOB values
fit.median <- list()
for (i in 1:length(fit.list)) {
    fit.median[[i]] <- median(fit.list[[i]]$err.rate[,
        "OOB"])
}
# Unlist the values and save err.obs.a.s.3
fit.median <- unlist(fit.median)
err.obs.a.s.3 <- data.frame(fit.median)
```

Year Randomized Data

```
# a.s.3 randomized
fit.list <- list()
for (i in 1:length(c(1:500))) {
    # for all items (i) in the list of chosen
    # length--change to number of iterations you want
    # to perform.
    cat("Repetition number ")  #Repetition number X will let us know that our loop is working.
    cat(i)
    cat("\n")
    # reload dataset
    df_250_1 <- read.csv("Cascade vs Coast 250 mmu and 1.csv")
    df_250_1 <- df_250_1[c(2, 7:952)]
    # make a new dataframe with the grouping variable.
    rand.col.1 <- data.frame(df_250_1$Year)
    names(rand.col.1)[1] <- "Year"
    rand.col.1$Year <- factor(rand.col.1$Year)
    is.factor(rand.col.1$Year)
    # must be true
```

```
    df_250_1$Year <- NULL
    # randomize the values in the grouping variable.
    rand.col.1 <- data.frame(rand.col.1[sample(nrow(rand.col.1)),
        ])
    # Recombine the randomized grouping variable with
    # the molecular abundance data.
    df <- cbind(rand.col.1, df_250_1)
    names(df)[1] <- "Year"
    df$Year <- factor(df$Year)
    is.factor(df$Year)  #this must be TRUE.

    # subset the data to obtain balanced class size.
    year.86 <- subset(df, Year == "86")
    year.87 <- subset(df, Year == "87")
    year.88 <- subset(df, Year == "88")
    year.86 <- year.86[sample((1:nrow(year.86)), 185,
        replace = FALSE), ]
    year.87 <- year.87[sample((1:nrow(year.87)), 185,
        replace = FALSE), ]
    year.88 <- year.88[sample((1:nrow(year.88)), 185,
        replace = FALSE), ]

    df.train <- rbind(year.86, year.87, year.88)

    # run randomForest
    fit.list[[i]] <- (fit.forest <- randomForest(Year ~
        ., data = df.train, importance = TRUE))  #Grows the forest

}  #end of for loop

fit.median <- list()
for (i in 1:length(fit.list)) {
    fit.median[[i]] <- median(fit.list[[i]]$err.rate[,
        "OOB"])
}
fit.median <- unlist(fit.median)
err.rand.a.s.3 <- data.frame(fit.median)
```

Year*Source Observed Data

Subset your data into a training and validation set. BUT MAKE SURE THAT THE CLASSES ARE BALANCED! For my data this required some calculations. The smallest class (Coast.86) in the grouping variable had 83 samples. 83 was set as the upper limit for each class. For this analysis, we are not interested in validating unknowns.

```
# a.r.s.6 observed
fit.list <- list()
for (i in 1:length(c(1:500))) {
    # for all items (i) in the list of chosen
    # length--change to number of iterations you want
    # to perform.
    cat("Repetition number ")  #Repetition number X will let us know that our loop is working.
    cat(i)
    cat("\n")
```

```
    # reload dataset
    df_250_1 <- read.csv("Cascade vs Coast 250 mmu and 1.csv")
    df <- df_250_1[c(3, 7:952)]
    # subset data for balanced classes.
    cas.86 <- subset(df, Reg.Sea == "Cascades.86")
    cas.87 <- subset(df, Reg.Sea == "Cascades.87")
    cas.88 <- subset(df, Reg.Sea == "Cascades.88")
    coa.86 <- subset(df, Reg.Sea == "Coast.86")
    coa.87 <- subset(df, Reg.Sea == "Coast.87")
    coa.88 <- subset(df, Reg.Sea == "Coast.88")
    cas.86 <- cas.86[sample((1:nrow(cas.86)), 83, replace = FALSE),
        ]
    cas.87 <- cas.87[sample((1:nrow(cas.87)), 83, replace = FALSE),
        ]
    cas.88 <- cas.88[sample((1:nrow(cas.88)), 83, replace = FALSE),
        ]
    coa.86 <- coa.86[sample((1:nrow(coa.86)), 83, replace = FALSE),
        ]
    coa.87 <- coa.87[sample((1:nrow(coa.87)), 83, replace = FALSE),
        ]
    coa.88 <- coa.88[sample((1:nrow(coa.88)), 83, replace = FALSE),
        ]
    df.train <- rbind(cas.86, cas.87, cas.88, coa.86,
        coa.87, coa.88)

    # run randomForest
    fit.list[[i]] <- (fit.forest <- randomForest(Reg.Sea ~
        ., data = df.train, importance = TRUE))  #Grows the forest
}  #end of for loop
fit.median <- list()
for (i in 1:length(fit.list)) {
    fit.median[[i]] <- median(fit.list[[i]]$err.rate[,
        "OOB"])
}
fit.median <- unlist(fit.median)
err.obs.a.r.s.6 <- data.frame(fit.median)
```

Year*Source Randomized Data

```
# a.r.s.6 randomized
fit.list <- list()
for (i in 1:length(c(1:500))) {
    # for all items (i) in the list of chosen
    # length--change to number of iterations you want
    # to perform.
    cat("Repetition number ")  #Repetition number X will let us know that our loop is working.
    cat(i)
    cat("\n")
    # reload the dataset
    df_250_1 <- read.csv("Cascade vs Coast 250 mmu and 1.csv")
    df_250_1 <- df_250_1[c(3, 7:952)]
    # randomize values in the grouping variable
    rand.col.1 <- data.frame(df_250_1$Reg.Sea)
```

```
    df_250_1$Reg.Sea <- NULL
    rand.col.1 <- data.frame(rand.col.1[sample(nrow(rand.col.1)),
        ])
    names(rand.col.1)[1] <- "Reg.Sea"
    df <- cbind(rand.col.1, df_250_1)
    # subset data for balanced classes.
    cas.86 <- subset(df, Reg.Sea == "Cascades.86")
    cas.87 <- subset(df, Reg.Sea == "Cascades.87")
    cas.88 <- subset(df, Reg.Sea == "Cascades.88")
    coa.86 <- subset(df, Reg.Sea == "Coast.86")
    coa.87 <- subset(df, Reg.Sea == "Coast.87")
    coa.88 <- subset(df, Reg.Sea == "Coast.88")
    cas.86 <- cas.86[sample((1:nrow(cas.86)), 83, replace = FALSE),
        ]
    cas.87 <- cas.87[sample((1:nrow(cas.87)), 83, replace = FALSE),
        ]
    cas.88 <- cas.88[sample((1:nrow(cas.88)), 83, replace = FALSE),
        ]
    coa.86 <- coa.86[sample((1:nrow(coa.86)), 83, replace = FALSE),
        ]
    coa.87 <- coa.87[sample((1:nrow(coa.87)), 83, replace = FALSE),
        ]
    coa.88 <- coa.88[sample((1:nrow(coa.88)), 83, replace = FALSE),
        ]
    df.train <- rbind(cas.86, cas.87, cas.88, coa.86,
        coa.87, coa.88)

    # run randomForest
    fit.list[[i]] <- (fit.forest <- randomForest(Reg.Sea ~
        ., data = df.train, importance = TRUE))  #Grows the forest

}  #end of for loop
fit.median <- list()
for (i in 1:length(fit.list)) {
    fit.median[[i]] <- median(fit.list[[i]]$err.rate[,
        "OOB"])
}
fit.median <- unlist(fit.median)
err.rand.a.r.s.6 <- data.frame(fit.median)
```

Save the data from above to your working directory for later analysis.

```
err.a.s.3 <- cbind(err.obs.a.s.3, err.rand.a.s.3)
names(err.a.s.3)[1] <- "Observed"
names(err.a.s.3)[2] <- "Randomized"
write.csv(err.a.s.3, "err.a.s.3.csv")
dens.a.s.3 <- err.a.s.3
temp <- err.a.s.3
temp[1] <- NULL
names(temp)[1] <- "OOB.med.err"
temp$group <- rep("Randomized", length(temp))
dens.a.s.3[2] <- NULL
names(dens.a.s.3)[1] <- "OOB.med.err"
dens.a.s.3$group <- rep("Observed", length(dens.a.s.3))
```

```
dens.a.s.3 <- rbind(dens.a.s.3, temp)
write.csv(dens.a.s.3, "dens.a.s.3.csv")

err.a.r.s.6 <- cbind(err.obs.a.r.s.6, err.rand.a.r.s.6)
names(err.a.r.s.6)[1] <- "Observed"
names(err.a.r.s.6)[2] <- "Randomized"
write.csv(err.a.r.s.6, "err.a.r.s.6.csv")
dens.a.r.s.6 <- err.a.r.s.6
temp <- err.a.r.s.6
temp[1] <- NULL
names(temp)[1] <- "OOB.med.err"
temp$group <- rep("Randomized", length(temp))
dens.a.r.s.6[2] <- NULL
names(dens.a.r.s.6)[1] <- "OOB.med.err"
dens.a.r.s.6$group <- rep("Observed", length(dens.a.r.s.6))
dens.a.r.s.6 <- rbind(dens.a.r.s.6, temp)
write.csv(dens.a.r.s.6, "dens.a.r.s.6.csv")
```

Results and Graphs

I have not provided these files. You will have to write the results from above as separate files and then bring them in from your own working directory.

```
# density plots data prep bring in v.r.2 data saved
# after random forest
v.r.2.errs <- read.csv("err.v.r.2.csv")
v.r.2.errs[1] <- NULL
v.r.2.dens <- read.csv("dens.v.r.2.csv")
v.r.2.dens[1] <- NULL

# bring in a.r.2 data saved after random forest
a.r.2.errs <- read.csv("err.a.r.2.csv")
a.r.2.errs[1] <- NULL
a.r.2.dens <- read.csv("dens.a.r.2.csv")
a.r.2.dens[1] <- NULL

# bring in a.s.3 data saved after random forest
a.s.3.errs <- read.csv("err.a.s.3.csv")
a.s.3.errs[1] <- NULL
a.s.3.dens <- read.csv("dens.a.s.3.csv")
a.s.3.dens[1] <- NULL

# bring in a.r.s.6 data saved after random forest
a.r.s.6.errs <- read.csv("err.a.r.s.6.csv")
a.r.s.6.errs[1] <- NULL
a.r.s.6.dens <- read.csv("dens.a.r.s.6.csv")
a.r.s.6.dens[1] <- NULL

# Calculate the compliment of OOB classification
# error or classification accuracy.

# Step 1: Convert to percentage
a.r.2.errs$Observed <- (a.r.2.errs$Observed * 100)
```

```
a.r.2.errs$Randomized <- (a.r.2.errs$Randomized * 100)
v.r.2.errs$Observed <- (v.r.2.errs$Observed * 100)
v.r.2.errs$Randomized <- (v.r.2.errs$Randomized * 100)
a.s.3.errs$Observed <- (a.s.3.errs$Observed * 100)
a.s.3.errs$Randomized <- (a.s.3.errs$Randomized * 100)
a.r.s.6.errs$Observed <- (a.r.s.6.errs$Observed * 100)
a.r.s.6.errs$Randomized <- (a.r.s.6.errs$Randomized *
    100)
a.r.2.dens$OOB.med.err <- (a.r.2.dens$OOB.med.err *
    100)
v.r.2.dens$OOB.med.err <- (v.r.2.dens$OOB.med.err *
    100)
a.s.3.dens$OOB.med.err <- (a.s.3.dens$OOB.med.err *
    100)
a.r.s.6.dens$OOB.med.err <- (a.r.s.6.dens$OOB.med.err *
    100)

# Step 2: Calculate compliment/accuracy
a.r.2.errs$Observed <- (100 - a.r.2.errs$Observed)
a.r.2.errs$Randomized <- (100 - a.r.2.errs$Randomized)
v.r.2.errs$Observed <- (100 - v.r.2.errs$Observed)
v.r.2.errs$Randomized <- (100 - v.r.2.errs$Randomized)
a.s.3.errs$Observed <- (100 - a.s.3.errs$Observed)
a.s.3.errs$Randomized <- (100 - a.s.3.errs$Randomized)
a.r.s.6.errs$Observed <- (100 - a.r.s.6.errs$Observed)
a.r.s.6.errs$Randomized <- (100 - a.r.s.6.errs$Randomized)
a.r.2.dens$OOB.med.err <- (100 - a.r.2.dens$OOB.med.err)
v.r.2.dens$OOB.med.err <- (100 - v.r.2.dens$OOB.med.err)
a.s.3.dens$OOB.med.err <- (100 - a.s.3.dens$OOB.med.err)
a.r.s.6.dens$OOB.med.err <- (100 - a.r.s.6.dens$OOB.med.err)

# Write out.

write.csv(a.r.2.errs, "a.r.2.acc.csv", row.names = FALSE)
write.csv(v.r.2.errs, "v.r.2.acc.csv", row.names = FALSE)
write.csv(a.s.3.errs, "a.s.3.acc.csv", row.names = FALSE)
write.csv(a.r.s.6.errs, "a.r.s.6.acc.csv", row.names = FALSE)
write.csv(a.r.2.dens, "a.r.2.dens.acc.csv", row.names = FALSE)
write.csv(v.r.2.dens, "v.r.2.dens.acc.csv", row.names = FALSE)
write.csv(a.s.3.dens, "a.s.3.dens.acc.csv", row.names = FALSE)
write.csv(a.r.s.6.dens, "a.r.s.6.dens.acc.csv", row.names = FALSE)
```

Calculate statistics and plot density graphs.

```
# Bring in those files you made above.  change the
# below to reflect your files and structure.

a.r.2.accs <- read.csv("a.r.2.acc_20170219.csv")
v.r.2.accs <- read.csv("v.r.2.acc_20170219.csv")
a.s.3.accs <- read.csv("a.s.3.acc_20170219.csv")
a.r.s.6.accs <- read.csv("a.r.s.6.acc_20170219.csv")
a.r.2.acc.dens <- read.csv("a.r.2.dens.acc_20170219.csv")
v.r.2.acc.dens <- read.csv("v.r.2.dens.acc_20170219.csv")
a.s.3.acc.dens <- read.csv("a.s.3.dens.acc_20170219.csv")
```

```
a.r.s.6.acc.dens <- read.csv("a.r.s.6.dens.acc_20170219.csv")

# a.r.2 density plot observed stats
mean.a.r.2.obs <- mean(a.r.2.accs$Observed)
error.a.r.2.obs <- qt(0.975, df = length(a.r.2.accs$Observed) -
    1) * sd(a.r.2.accs$Observed)/sqrt(length(a.r.2.accs$Observed))
a.r.2.obs.left <- mean(a.r.2.accs$Observed) - error.a.r.2.obs
a.r.2.obs.right <- mean(a.r.2.accs$Observed) + error.a.r.2.obs
a.r.2.obs.random.forest.stats <- data.frame(cbind(mean.a.r.2.obs,
    error.a.r.2.obs, a.r.2.obs.left, a.r.2.obs.right))
names(a.r.2.obs.random.forest.stats) <- c("mean", "se",
    "lower", "upper")
a.r.2.obs.random.forest.stats$format <- c("observed")
# randomized stats
mean.a.r.2.rand <- mean(a.r.2.accs$Randomized)
error.a.r.2.rand <- qt(0.975, df = length(a.r.2.accs$Randomized) -
    1) * sd(a.r.2.accs$Randomized)/sqrt(length(a.r.2.accs$Randomized))
a.r.2.rand.left <- mean(a.r.2.accs$Randomized) - error.a.r.2.rand
a.r.2.rand.right <- mean(a.r.2.accs$Randomized) + error.a.r.2.rand
a.r.2.rand.random.forest.stats <- data.frame(cbind(mean.a.r.2.rand,
    error.a.r.2.rand, a.r.2.rand.left, a.r.2.rand.right))
names(a.r.2.rand.random.forest.stats) <- c("mean",
    "se", "lower", "upper")
a.r.2.rand.random.forest.stats$format <- c("randomized")
a.r.2.random.forest.stats <- rbind(a.r.2.obs.random.forest.stats,
    a.r.2.rand.random.forest.stats)
a.r.2.random.forest.stats$model <- c("source.indiv",
    "source.indiv")
# write.csv(a.r.2.random.forest.stats,'path/filename.csv',row.names=FALSE)
```

Code for plot (Fig. S1.1 C).

```
RFInd <- ggplot(a.r.2.acc.dens, aes(x = OOB.med.err,
    fill = group)) + geom_density(alpha = 0.7) + scale_fill_manual(values = c("grey80",
    "grey50"), labels = c("Observed Data", "Randomized Data")) +
    geom_vline(xintercept = mean.a.r.2.rand, colour = "black") +
    geom_vline(xintercept = mean.a.r.2.obs, colour = "blue") +
    theme_classic() + theme_bw() + scale_x_continuous(limits = c(0,
    100)) + xlab("Classification Accuracy (%)") + ylab("Kernal Density Estimate") +
    ggtitle("SourceINDIV") + theme(panel.grid.major = element_blank(),
    panel.grid.minor = element_blank(), plot.title = element_text(hjust = 0),
    legend.justification = c(1, 1), legend.position = c(0.5,
        1), legend.title = element_blank(), legend.background = element_blank(),
    legend.key.size = unit(0.4, "cm"), legend.text = element_text(size = 8),
    axis.title = element_text(size = 8), legend.key = element_rect(color = "white"))
```

```
# v.r.2 density plot observed stats
mean.v.r.2.obs <- mean(v.r.2.accs$Observed)
error.v.r.2.obs <- qt(0.975, df = length(v.r.2.accs$Observed) -
    1) * sd(v.r.2.accs$Observed)/sqrt(length(v.r.2.accs$Observed))
v.r.2.obs.left <- mean(v.r.2.accs$Observed) - error.a.r.2.obs
v.r.2.obs.right <- mean(v.r.2.accs$Observed) + error.a.r.2.obs
v.r.2.obs.random.forest.stats <- data.frame(cbind(mean.v.r.2.obs,
    error.v.r.2.obs, v.r.2.obs.left, v.r.2.obs.right))
names(v.r.2.obs.random.forest.stats) <- c("mean", "se",
```

```
    "lower", "upper")
v.r.2.obs.random.forest.stats$format <- c("observed")
# randomized stats
mean.v.r.2.rand <- mean(v.r.2.accs$Randomized)
error.v.r.2.rand <- qt(0.975, df = length(v.r.2.accs$Randomized) -
    1) * sd(v.r.2.accs$Randomized)/sqrt(length(v.r.2.accs$Randomized))
v.r.2.rand.left <- mean(v.r.2.accs$Randomized) - error.v.r.2.rand
v.r.2.rand.right <- mean(v.r.2.accs$Randomized) + error.v.r.2.rand
v.r.2.rand.random.forest.stats <- data.frame(cbind(mean.v.r.2.rand,
    error.v.r.2.rand, v.r.2.rand.left, v.r.2.rand.right))
names(v.r.2.rand.random.forest.stats) <- c("mean",
    "se", "lower", "upper")
v.r.2.rand.random.forest.stats$format <- c("randomized")
v.r.2.random.forest.stats <- rbind(v.r.2.obs.random.forest.stats,
    v.r.2.rand.random.forest.stats)
v.r.2.random.forest.stats$model <- c("source.mean",
    "source.mean")
# write.csv(v.r.2.random.forest.stats,'path/filename.csv',row.names=FALSE)
```

Code for plot (Fig. S1.1 D).

```
RFMean <- ggplot(v.r.2.acc.dens, aes(x = OOB.med.err,
    fill = group)) + geom_density(alpha = 0.7) + scale_fill_manual(values = c("grey80",
    "grey50"), labels = c("Observed Data", "Randomized Data")) +
    geom_vline(xintercept = mean.v.r.2.rand, colour = "black") +
    geom_vline(xintercept = mean.v.r.2.obs, colour = "blue") +
    theme_classic() + theme_bw() + scale_x_continuous(limits = c(0,
    100)) + xlab("Classification Accuracy (%)") + ylab("Kernal Density Estimate") +
    ggtitle("SourceMEAN") + theme(panel.grid.major = element_blank(),
    panel.grid.minor = element_blank(), plot.title = element_text(hjust = 0),
    legend.justification = c(1, 1), legend.position = c(0.5,
        1), legend.title = element_blank(), legend.background = element_blank(),
    legend.key.size = unit(0.4, "cm"), legend.text = element_text(size = 8),
    axis.title = element_text(size = 8), legend.key = element_rect(color = "white"))
```

```
# a.s.3 density plot observed stats
mean.a.s.3.obs <- mean(a.s.3.accs$Observed)
error.a.s.3.obs <- qt(0.975, df = length(a.s.3.accs$Observed) -
    1) * sd(a.s.3.accs$Observed)/sqrt(length(a.s.3.accs$Observed))
a.s.3.obs.left <- mean(a.s.3.accs$Observed) - error.a.s.3.obs
a.s.3.obs.right <- mean(a.s.3.accs$Observed) + error.a.s.3.obs
a.s.3.obs.random.forest.stats <- data.frame(cbind(mean.a.s.3.obs,
    error.a.s.3.obs, a.s.3.obs.left, a.s.3.obs.right))
names(a.s.3.obs.random.forest.stats) <- c("mean", "se",
    "lower", "upper")
a.s.3.obs.random.forest.stats$format <- c("observed")
# randomized stats
mean.a.s.3.rand <- mean(a.s.3.accs$Randomized)
error.a.s.3.rand <- qt(0.975, df = length(a.s.3.accs$Randomized) -
    1) * sd(a.s.3.accs$Randomized)/sqrt(length(a.s.3.accs$Randomized))
a.s.3.rand.left <- mean(a.s.3.accs$Randomized) - error.a.s.3.rand
a.s.3.rand.right <- mean(a.s.3.accs$Randomized) + error.a.s.3.rand
a.s.3.rand.random.forest.stats <- data.frame(cbind(mean.a.s.3.rand,
    error.a.s.3.rand, a.s.3.rand.left, a.s.3.rand.right))
names(a.s.3.rand.random.forest.stats) <- c("mean",
```

```
    "se", "lower", "upper")
a.s.3.rand.random.forest.stats$format <- c("randomized")
a.s.3.random.forest.stats <- rbind(a.s.3.obs.random.forest.stats,
    a.s.3.rand.random.forest.stats)
a.s.3.random.forest.stats$model <- c("year", "year")
# write.csv(a.s.3.random.forest.stats,'path/filename.csv',row.names=FALSE)
```

Code for plot (Fig. S1.1 A).

```
RFY <- ggplot(a.s.3.acc.dens, aes(x = OOB.med.err,
    fill = group)) + geom_density(alpha = 0.7) + scale_fill_manual(values = c("grey80",
    "grey50"), labels = c("Observed Data", "Randomized Data")) +
    geom_vline(xintercept = mean.a.s.3.rand, colour = "black") +
    geom_vline(xintercept = mean.a.s.3.obs, colour = "blue") +
    theme_classic() + theme_bw() + scale_x_continuous(limits = c(0,
    100)) + xlab("Classification Accuracy (%)") + ylab("Kernal Density Estimate") +
    ggtitle("Year") + theme(panel.grid.major = element_blank(),
    panel.grid.minor = element_blank(), plot.title = element_text(hjust = 0),
    legend.justification = c(1, 1), legend.position = c(1,
        1), legend.title = element_blank(), legend.background = element_blank(),
    legend.key.size = unit(0.4, "cm"), legend.text = element_text(size = 8),
    axis.title = element_text(size = 8), legend.key = element_rect(color = "white"))

# a.r.s.6 density plot observed stats
mean.a.r.s.6.obs <- mean(a.r.s.6.accs$Observed)
error.a.r.s.6.obs <- qt(0.975, df = length(a.r.s.6.accs$Observed) -
    1) * sd(a.r.s.6.accs$Observed)/sqrt(length(a.r.s.6.accs$Observed))
a.r.s.6.obs.left <- mean(a.r.s.6.accs$Observed) - error.a.r.s.6.obs
a.r.s.6.obs.right <- mean(a.r.s.6.accs$Observed) +
    error.a.r.s.6.obs
a.r.s.6.obs.random.forest.stats <- data.frame(cbind(mean.a.r.s.6.obs,
    error.a.r.s.6.obs, a.r.s.6.obs.left, a.r.s.6.obs.right))
names(a.r.s.6.obs.random.forest.stats) <- c("mean",
    "se", "lower", "upper")
a.r.s.6.obs.random.forest.stats$format <- c("observed")
# randomized stats
mean.a.r.s.6.rand <- mean(a.r.s.6.accs$Randomized)
error.a.r.s.6.rand <- qt(0.975, df = length(a.r.s.6.accs$Randomized) -
    1) * sd(a.r.s.6.accs$Randomized)/sqrt(length(a.r.s.6.accs$Randomized))
a.r.s.6.rand.left <- mean(a.r.s.6.accs$Randomized) -
    error.a.r.s.6.rand
a.r.s.6.rand.right <- mean(a.r.s.6.accs$Randomized) +
    error.a.r.s.6.rand
a.r.s.6.rand.random.forest.stats <- data.frame(cbind(mean.a.r.s.6.rand,
    error.a.r.s.6.rand, a.r.s.6.rand.left, a.r.s.6.rand.right))
names(a.r.s.6.rand.random.forest.stats) <- c("mean",
    "se", "lower", "upper")
a.r.s.6.rand.random.forest.stats$format <- c("randomized")
a.r.s.6.random.forest.stats <- rbind(a.r.s.6.obs.random.forest.stats,
    a.r.s.6.rand.random.forest.stats)
a.r.s.6.random.forest.stats$model <- c("year.source",
    "year.source")
# write.csv(a.r.s.6.random.forest.stats,'path/filename.csv',row.names=FALSE)
```

Code for plot (Fig. S1.1 B).

```
RFYR <- ggplot(a.r.s.6.acc.dens, aes(x = OOB.med.err,
```

```
    fill = group)) + geom_density(alpha = 0.7) + scale_fill_manual(values = c("grey80",
    "grey50"), labels = c("Observed Data", "Randomized Data")) +
    geom_vline(xintercept = mean.a.r.s.6.rand, colour = "black") +
    geom_vline(xintercept = mean.a.r.s.6.obs, colour = "blue") +
    theme_classic() + theme_bw() + scale_x_continuous(limits = c(0,
    100)) + xlab("Classification Accuracy (%)") + ylab("Kernal Density Estimate") +
    ggtitle("Year*Source") + theme(panel.grid.major = element_blank(),
    panel.grid.minor = element_blank(), plot.title = element_text(hjust = 0),
    legend.justification = c(1, 1), legend.position = c(1,
        1), legend.title = element_blank(), legend.background = element_blank(),
    legend.key.size = unit(0.4, "cm"), legend.text = element_text(size = 8),
    axis.title = element_text(size = 8), legend.key = element_rect(color = "white"))

# bring together stats from all models
random.forest.stats <- data.frame(rbind(a.s.3.random.forest.stats,
    a.r.s.6.random.forest.stats, v.r.2.random.forest.stats,
    a.r.2.random.forest.stats))
# write.csv(random.forest.stats,'Revision_1_datasets/random.forest.stats.csv',
# row.names = FALSE)
random.forest.stats
```

```
##       mean         se    lower    upper     format        model
## 1 24.50172 0.09435472 24.40737 24.59608   observed         year
## 2 32.88643 0.18097771 32.70546 33.06741 randomized         year
## 3 16.01527 0.10486770 15.91040 16.12014   observed  year.source
## 4 16.19120 0.15733344 16.03386 16.34853 randomized  year.source
## 5 70.09133 0.18578071 69.99959 70.18308   observed  source.mean
## 6 48.91364 0.43004359 48.48359 49.34368 randomized  source.mean
## 7 75.72991 0.09174699 75.63816 75.82166   observed source.indiv
## 8 49.77670 0.24042774 49.53627 50.01713 randomized source.indiv
```

```
grid.arrange(RFY,RFYR,RFInd,RFMean,ncol=2)
```

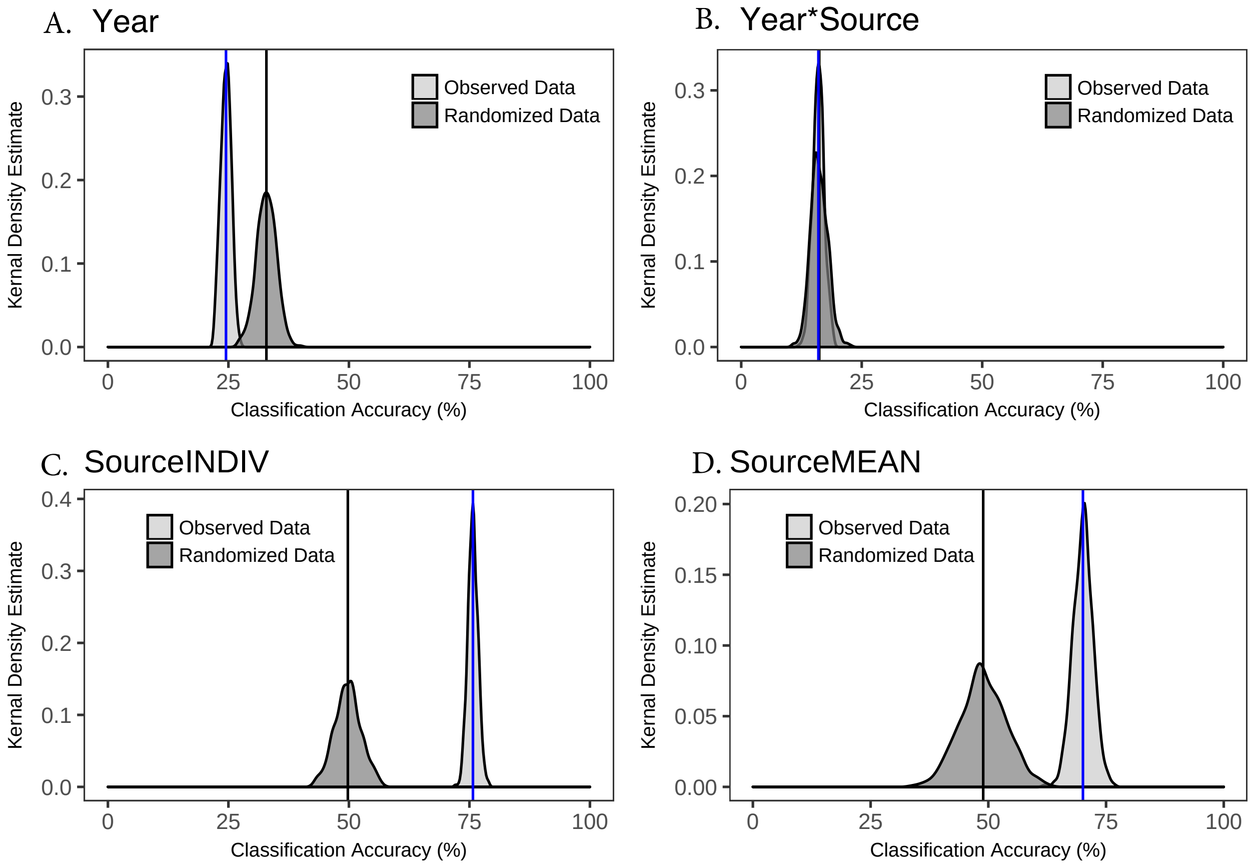

Figure S1.1. Distributions of the classification accuracies from random forests. Dark grey distributions were generated from randomized data, and light grey distributions were generated from observed data. Blue lines indicate the estimated mean classification accuracy for observed data, and black lines indicate the estimated mean classification accuracy for randomized data. Classification accuracies are shown for (A) Year, (B) Year*Source, (C) SourceINDIV, and (D) SourceMEAN models.

ROC Curves showing model performance (SourceINDIV and SourceMEAN). These analyses can only be performed for binary classifications only.

```
#ROCs
#v.r.2 for ROC
#change the below to reflect your files and structure.
roc.v.r.2_20170218<-read.csv("roc.total.obs.v.r.2_20170218.csv")
roc.v.r.2_20170218$replicate<-factor(roc.v.r.2_20170218$replicate)
#a.r.2 for ROC
roc.a.r.2_20170218<-read.csv("roc.total.obs.a.r.2_20170218.csv")
roc.a.r.2_20170218$replicate<-factor(roc.a.r.2_20170218$replicate)
#ROC Graphs
```

Code for plot (Fig. S1.2 A)

```
roc.v.r.2_20170218_plot<-ggplot(roc.v.r.2_20170218,
                                 aes(x=x_axis, y=y_axis)) +
  geom_line(aes(color=replicate), alpha=0.2)+
  geom_smooth(colour="darkred", method="auto") +
  xlab("False Positive Rate")+
  ylab("True Positive Rate")+
  scale_color_grey()+
  ggtitle("SourceMEAN")+
  theme_bw()+
  theme(
```

```
    legend.position = "none",
    plot.title = element_text(hjust = 0),
    panel.grid.major = element_blank(),
    panel.grid.minor = element_blank(),
    axis.title=element_text(size=8),
    axis.text.x=element_text(size=8),
    axis.text.y=element_text(size=8))
```

Code for plot (Fig. S1.2 B)

```
roc.a.r.2_20170218_plot<-ggplot(roc.a.r.2_20170218,
                                aes(x=x_axis, y=y_axis)) +
  geom_line(aes(color=replicate), alpha=0.2)+
  geom_smooth(colour="midnightblue", method="auto") +
  xlab("False Positive Rate")+
  ylab("True Positive Rate")+
  scale_color_grey()+
  ggtitle("SourceINDIV")+
  theme_bw()+
  theme(
    legend.position = "none",
    plot.title = element_text(hjust = 0),
    panel.grid.major = element_blank(),
    panel.grid.minor = element_blank(),
    axis.title=element_text(size=8),
    axis.text.x=element_text(size=8),
    axis.text.y=element_text(size=8))

vr2<-roc.v.r.2_20170218
vr2.group<-data.frame(rep("vr2",18646))#you might need to change length
names(vr2.group)[1]<-"group"
vr2<-cbind(vr2,vr2.group)
ar2<-roc.a.r.2_20170218
ar2.group<-data.frame(rep("ar2",49833))#you might need to change length
names(ar2.group)[1]<-"group"
ar2<-cbind(ar2,ar2.group)
combined.roc_20170218<-rbind(ar2,vr2)
write.csv(combined.roc_20170218, "combined.roc_20170218.csv", row.names = FALSE)
```

Code for plot (Fig. S1.2 C)

```
combined.roc.plot_20170218<-ggplot(combined.roc_20170218, aes(x=x_axis, y=y_axis, color=factor(group)))
  geom_blank() +
  stat_smooth(aes(colour = factor(group)),se=FALSE, method="auto") +
  theme_bw()+theme(legend.position = "none")+
  xlab("False Positive Rate")+
  ylab("True Positive Rate")+scale_color_manual(values=c("midnightblue", "darkred"))+
  ggtitle("Comparison")+
  theme(
    panel.grid.major = element_blank(),
    panel.grid.minor = element_blank(),
    plot.title = element_text(hjust = 0),
    axis.title=element_text(size=8),
    axis.text.x=element_text(size=8),
    axis.text.y=element_text(size=8))
```

Combined ROC Figure

```
grid.arrange(roc.v.r.2_20170218_plot, roc.a.r.2_20170218_plot, combined.roc.plot_20170218, ncol=1)
```

```
## `geom_smooth()` using method = 'gam'
## `geom_smooth()` using method = 'gam'
## `geom_smooth()` using method = 'gam'
```

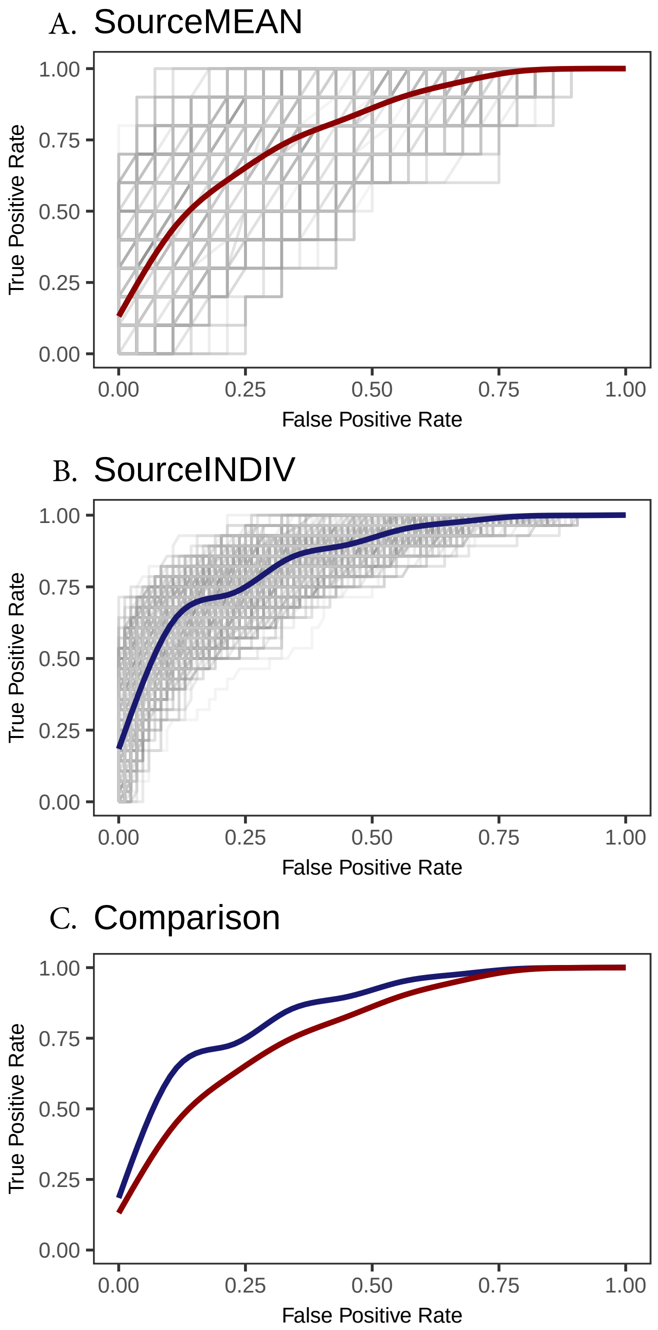

Figure S1.2. ROC curves generated for 500 Random Forests by predicting the class membership of each sample in a validation set. The x-axis is the false positive rate and the y-axis is true positive rate. Grey lines indicate individual ROC curves from each of the 500 iterations. Colored lines indicate the estimated mean ROC curve generated with a generalized additive model and a cubic spline. (A) ROC plots for the SourceMEAN model, (B) ROC plots for the SourceINDIV model, and (C) superimposed mean ROC curves for SourceINDIV (blue) and SourceMEAN (red) models.

Append the following code after the random forest analysis contained in fit.list to obtain the OOB error rates for each class in the grouping variable. This code will not be evaluated here.

```
fit.median.cas <- list()
for (i in 1:length(fit.list)) {
    fit.median.cas[[i]] <- median(fit.list[[i]]$err.rate[,
```

```
        "Cascades"])
}
fit.median.coa <- list()
for (i in 1:length(fit.list)) {
    fit.median.coa[[i]] <- median(fit.list[[i]]$err.rate[,
        "Coast"])
}
# Unlisting the values
fit.median.coa <- unlist(fit.median.coa)
a.r.2.err.coa <- data.frame(fit.median.coa)
fit.median.cas <- unlist(fit.median.cas)
a.r.2.err.cas <- data.frame(fit.median.cas)

a.r.2.err.cas$group <- rep("Cascades", length(a.r.2.err.cas))
names(a.r.2.err.cas)[1] <- "class.err"

a.r.2.err.coa$group <- rep("Coast", length(a.r.2.err.coa))
names(a.r.2.err.coa)[1] <- "class.err"

a.r.2.err.rates.by.class <- rbind(a.r.2.err.cas, a.r.2.err.coa)
# convert to accuracy
a.r.2.err.rates.by.class$class.err <- (a.r.2.err.rates.by.class$class.err) *
    100
a.r.2.err.rates.by.class$class.err <- 100 - (a.r.2.err.rates.by.class$class.err)  #write this out write.
```

Graph and paired t-test. Bring in the files from the code directly above.

```
# asymmetry analysis v.r.2 SourceMEAN model bring
# in your files
v.r.2.err.rates.by.class <- read.csv("v.r.2.err.rates.by.class.csv")
```

Code for plot (Fig. S1.3 B)

```
v.r.2.err.rates.by.class_plot <- ggplot(v.r.2.err.rates.by.class,
   aes(x = group, y = class.err, fill = group, alpha = 0.5)) +
   geom_boxplot(size = 0.3, outlier.colour = "black",
       outlier.shape = 1) + scale_fill_manual(values = c("red",
   "blue")) + theme_bw() + theme(panel.grid.major = element_blank(),
   panel.grid.minor = element_blank(), plot.title = element_text(hjust = 0),
   legend.position = "none", axis.title = element_text(size = 8),
   axis.text = element_text(size = 8)) + xlab("Class") +
   ylab("Classification Accuracy (%)") + ggtitle("SourceMEAN")
```

```
v.r.2.err.rates.by.class_test <- t.test(class.err ~
    group, data = v.r.2.err.rates.by.class, paired = TRUE)

# asymmetry analysis a.r.2 SourceINDIV model bring
# in your files
a.r.2.err.rates.by.class <- read.csv("a.r.2.err.rates.by.class_85.csv")
```

Code for plot (Fig. S1.3 A)

```
a.r.2.err.rates.by.class_plot <- ggplot(a.r.2.err.rates.by.class,
    aes(x = group, y = class.err, fill = group, alpha = 0.5)) +
    geom_boxplot(size = 0.3, outlier.colour = "black",
        outlier.shape = 1) + scale_fill_manual(values = c("red",
    "blue")) + theme_bw() + theme(panel.grid.major = element_blank(),
    panel.grid.minor = element_blank(), plot.title = element_text(hjust = 0),
    legend.position = "none", axis.title = element_text(size = 8),
    axis.text = element_text(size = 8)) + xlab("Class") +
    ylab("Classification Accuracy (%)") + ggtitle("SourceINDIV")

a.r.2.err.rates.by.class_test <- t.test(class.err ~
    group, data = a.r.2.err.rates.by.class, paired = TRUE)

# Show results of t.test
a.r.2.err.rates.by.class_test
```

```
##
##  Paired t-test
##
## data:  class.err by group
## t = -59.915, df = 499, p-value < 2.2e-16
## alternative hypothesis: true difference in means is not equal to 0
## 95 percent confidence interval:
##  -5.967759 -5.588795
## sample estimates:
## mean of the differences
##               -5.778277
```

```
v.r.2.err.rates.by.class_test
```

```
##
##  Paired t-test
##
## data:  class.err by group
## t = -48.632, df = 499, p-value < 2.2e-16
## alternative hypothesis: true difference in means is not equal to 0
## 95 percent confidence interval:
##  -9.468792 -8.733423
## sample estimates:
## mean of the differences
##               -9.101107
```

```
grid.arrange(a.r.2.err.rates.by.class_plot,v.r.2.err.rates.by.class_plot,ncol=1)
```

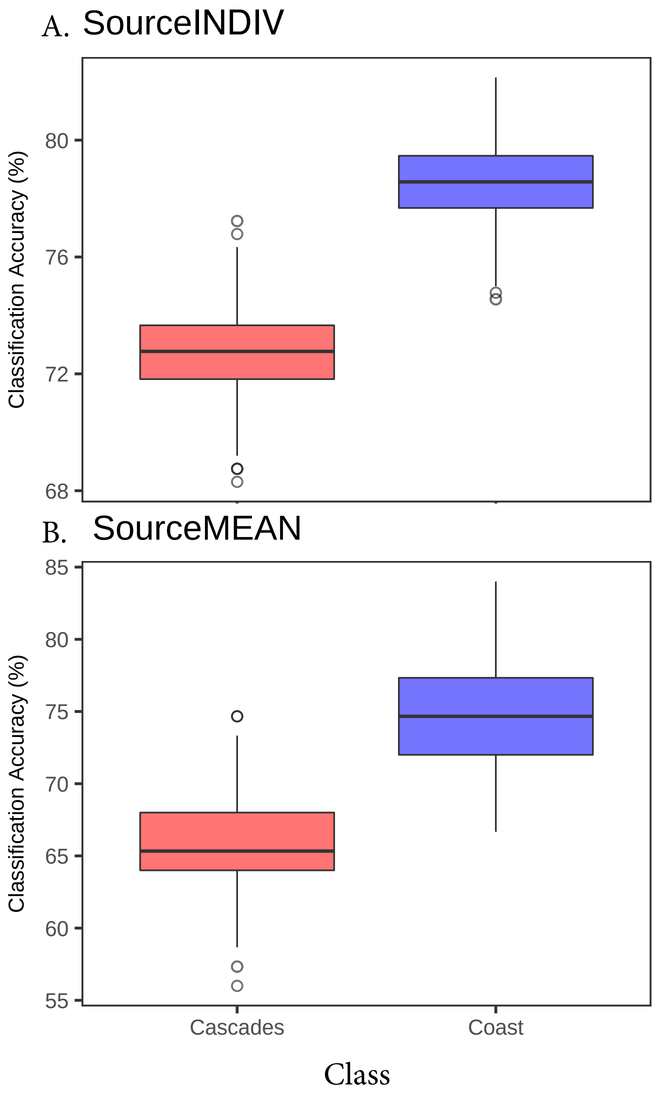

Figure S1.3. Boxplot showing the difference in random forest classification accuracies for the Cascade Range class and Coast Range class based on 500 iterations of random forest analysis each with 500 classification trees. (A) Classification accuracies for Cascade and Coast classes based on 560 individual spectra (SourceINDIV model) and (B) Classification accuracies for Cascade and Coast classes based on 188 mean spectra (SourceMEAN model).

The results of the above analysis WILL CHANGE if class size is not balanced. Classification will favor the majority class.
