# Supplementary material for: Source identification of western Oregon Douglas-fir wood cores using mass spectrometry and random forest classification
Source: Appl Plant Sci. 2017 May 12;5(5):apps.1600158. doi: 10.3732/apps.1600158 (PMC5435404; doi:10.3732/apps.1600158)
Supplement: Supplementary file 4 [file apps.1600158_s4.docx]

Appendix S4. Putative identities for 65 of the 946 putative ions detected among our samples via DART-TOFMS. Identities were approximated in Mass Mountaineer by comparing the mass-to-charge ratio of each molecule to a list of molecules identified in *Pinus* and *Pseudotsuga*. Provided are names that have been used to describe the molecules, their molecular formula, their mass-to-charge ratio, and the species from which they were identified.

| Molecule name | Molecular formula | Mass (*m/z*) | Species |
| --- | --- | --- | --- |
| 2-Phenylethanol | C_8_H_10_O | 123.113 | *Pinus massoniana* |
| p-Cymene | C_10_H_14_ | 135.1017 | *Pinus densiflora* |
| (+)-S-Carvone, 4(10)-Thujene, gamma-Terpinene, alpha-Phellandrene, Camphene, Myrcene, Limonene, beta-Pinene, alpha-Pinene, Teresantanane, Terpinolene, (E)-beta-Ocimene, (+/-)-beta-Phellandrene, alpha-Terpinene, 3-Carene, (-)-Sabinene, (-)-beta-Phellandrene, Dipentene, m-Mentha-1, 3(8)-diene | C_10_H_16_ | 136.0618 | *Pinus ayacahuite, P. balfouriana, P. banksiana, P. bungeana, P. canariensis, P. contorta, P. coulteri, P. echinata, P. eldarica, P. excelsa, P. formosana, P. grandis, P. halepensis, P. koraiensis, P. krempfii, P. lambertiana, P. luchuensis, P. massoniana, P. montana*, *P. morrisonicola*, *P. muricata*, *P. pallasiana*, *P. palustris*, *P. sibirica*, *P. sosnowskyi*, *P. strobus*, *P. sylvestris*, *P. taeda*, *P. thunbergii*, *Pseudotsuga japonica*, *P. menziesii*, *P. wilsoniana* |
| Anisaldehyde | C_8_H_8_O_2_ | 136.0618 | *Pinus thunbergii* |
| Pinidine | C_9_H_17_N | 139.0363 | *Pinus strobus*, *P. sylvestris*, *Pseudotsuga wilsoniana*, *P. menziesii* |
| Indole-3-carboxaldehyde | C_9_H_7_NO | 145.04871 | *Pseudotsuga menziesii* |
| Estragol | C_10_H_12_O | 149.11681 | *Pinus massoniana*, *P. mugo* |
| (R)-(+)-Verbenone | C_10_H_14_O | 150.07539 | *Pinus contorta* |
| (+)-Camphor, (+)-fenchone, isothujone, trans-thujone, R-(+)-trans-verbenol | C_10_H_16_O | 153.12711 | *Pinus cembra*, *P. contorta*, *P. elliottii*, *P. halepensis*, *P. kochiana*, *P. longifolia*, *P. palustris* |
| Vanillin | C_8_H_8_O_3_ | 153.12711 | *Pinus sibirica* |
| (R)-linalool, 1, 8-Cineole, Citronellal, (+)-m-Menth-6-en-8-ol, m-Menth-1-en-8-ol, p-Menth-4(8)-en-1-ol, trans-m-Menth-8-en-1-ol, borneol | C_10_H_18_O | 155.0592 | *Pinus contorta, P. halepensis, P. koraiensis, P. morrisonicola, P. occidentalis, P. pallasiana*, *P. sabiniana* |
| Indole-3-ethanol | C_10_H_11_NO | 161.118 | *Pseudotsuga menziesii* |
| Indole-3-carboxylic acid | C_9_H_7_NO_2_ | 161.118 | *Pseudotsuga menziesii* |
| Indole-3-acetic acid | C_10_H_9_NO_2_ | 175.11121 | *Pinus halepensis*, *P. laricio*, *P. ponderosa*, *P. sabiniana*, *P. sylvestris*, *Pseudotsuga wilsoniana* |
| Methyleugenol | C_11_H_14_O_2_ | 179.0748 | *Pinus morrisonicola* |
| Coniferyl alcohol | C_10_H_12_O_3_ | 180.0845 | *Pinus monticola*, *Pseudotsuga menziesii* |
| Stilbene | C_14_H_12_ | 180.0845 | *Pinus parviflora*, *Pseudotsuga wilsoniana* |
| Methyl indole-3-acetate | C_11_H_11_NO_2_ | 189.155 | *Pinus sylvestris*, *Pseudotsuga wilsoniana* |
| Ferulic acid | C_10_H_10_O_4_ | 194.1263 | *Pinus halepensis*, *P. sylvestris* |
| D-Pinitol | C_7_H_14_O_6_ | 194.1263 | *Pinus halepensis*, *P. sylvestris* |
| Bornyl acetate | C_12_H_20_O_2_ | 196.16991 | *Pinus caribaea, P. elliottii, P. excelsa, P. halepensis, P. laricio, P. muricata, P. palustris, P. ponderosa, P. sibirica, P. strobus, P. sylvestris, P. taeda, Pseudotsuga menziesii*, *P. wilsoniana* |
| N6-(delta2-Isopentenyl)adenine | C_10_H_13_N_5_ | 203.1796 | *Pseudotsuga wilsoniana* |
| (R)-(-)-alpha-Curcumene | C_15_H_22_ | 203.1796 | *Pinus contorta* |
| (E)-Caryophyllene, Humulene, Longifolene, delta-Cadinene, alpha-muurolene, beta-gurjunene, (Z)-beta-farnesene, (E)-beta-bourbonene, (-)-isocaryophyllene, (-)-zingiberene, copaene, cyclohexane, beta-sesquiphellandrene, gamma-muurolene, gamma-cadinene, longicyclene, (-)-germacrene D | C_15_H_24_ | 205.0872 | *Pinus cembra*, *P. contorta*, *P. coulteri*, *P. eldarica*, *P. elliottii*, *P. excelsa*, *P. flexilis*, *P. formosana*, *P. grandis*, *P. halepensis*, *P. leiophylla*, *P. longifolia*, *P. massoniana*, *P. monticola*, *P. palustris*, *P. parviflora*, *P. pinaster* |
| 4-Chloroindole-3-acetic acid | C_10_H_8_ClNO_2_ | 209.19 | *Pinus muricata*, *Pseudotsuga menziesii* |
| Pinosylvin | C_14_H_12_O_2_ | 212.2058 | *Pinus sibirica*, *P. sosnowskyi*, *P. strobus*, *P. sylvestris*, *Pseudotsuga menziesii*, *P. wilsoniana* |
| Omega-Hydroxydodecanoic acid | C_12_H_24_O_3_ | 217.1687 | *Pinus koraiensis* |
| (-)-beta-Caryophyllene epoxide,(-)-Humulene epoxide II | C_15_H_24_O | 220.18089 | *Pinus contorta* |
| delta-Cadinol, guaiol, elemol, copaborneol, (-)-alpha-cadinol, nerolidol | C_15_H_26_O | 222.2213 | *Pinus cembroides*, *P. clausa*, *P. densata*, *P. eldarica*, *P. elliottii*, *P. halepensis*, *P. lambertiana*, *P. radiata*, *P. rigida*, *P. sabiniana*, *P. sylvestris*, *Pseudotsuga wilsoniana* |
| 4-Chloroindole-3-acetic acid methyl ester | C_11_H_10_ClNO_2_ | 223.10049 | *Pinus muricata*, *Pseudotsuga menziesii* |
| Pinosylvin methyl ether | C_15_H_14_O_2_ | 227.17841 | *Pinus albicaulis, P. aristata, P. armandii, P. attenuata, P. balfouriana, P. banksiana, P. cembra, P. cembroides, P. clausa, P. contorta, P. densiflora, P. excelsa, P. gerardiana, P. grandis, P. halepensis, P. jeffreyi, P. kesiya, P. khasya, P. kochiana, P. koraiensis, P. monticola*, *P. occidentalis*, *P. parviflora*, *P. pentaphylla* |
| Resveratrol | C_14_H_12_O_3_ | 229.1907 | *Pinus krempfii*, *P. montana* |
| Dihydropinosylvin monomethyl ether | C_15_H_16_O_2_ | 229.1907 | *Pinus albicaulis*, *P. aristata*, *P. armandii*, *P. attenuata*, *P. canariensis*, *P. cembra*, *P. formosana*, *P. grandis*, *P. halepensis*, *P. jeffreyi*, *P. kochiana*, *P. montezumae* |
| ar-Pseudotsugonal | C_15_H_20_O_2_ | 233.1608 | *Pinus pinaster* |
| Atlantolone, pseudotsugonal | C_15_H_24_O_2_ | 236.17751 | *Pinus pumila*, *P. radiata*, *Pseudotsuga wilsoniana* |
| Dihydropseudotsugonal | C_15_H_26_O_2_ | 238.19279 | *Pinus pinea*, *Pseudotsuga wilsoniana* |
| Dihydropseudotsugonol | C_15_H_28_O_2_ | 241.20239 | *Pinus pinea* |
| (+/-)-ar-Todomatuic acid | C_15_H_20_O_3_ | 249.15759 | *Pinus pinaster* |
| Chrysin | C_15_H_10_O_4_ | 254.16541 | *Pinus aristata, P. cembra, P. halepensis, P. lumholtzii, P. resinosa, P. sibirica, P. sylvestris, P. taeda, Pseudotsuga wilsoniana* |
| Pinocembrin | C_15_H_12_O_4_ | 256.20139 | *Pinus banksiana*, *P. massoniana*, *P. sabiniana*, *P. slyvestris*, *P. virginiana*, *Pseudotsuga wilsoniana* |
| Ampullicin, isoampullicin | C_15_H_17_NO_3_ | 259.2406 | *Pseudotsuga wilsoniana* |
| Epijuvabione | C_16_H_26_O_3_ | 266.17599 | *Pinus pungens*, *Pseudotsuga wilsoniana* |
| Strobochrysin, tectochrysin | C_16_H_12_O_4_ | 269.21591 | *Pinus montana*, *P. monticola* |
| Strobopinin, (2S)-pinocembrin, 8-methylpinocembrin | C_16_H_14_O_4_ | 270.20721 | *Pinus halepensis*, *P. longifolia*, *P. lumholtzii*, *P. massoniana*, *P. monticola*, *P. sibirica*, *P. strobus*, *P. taeda*, *P. wallichiana*, *Pseudotsuga menziesii*, *P. wilsoniana* |
| Bietatriene | C_20_H_30_ | 270.20721 | *Pinus halepensis*, *P. sylvestris* |
| Pinobanksin, naringenin | C_15_H_12_O_5_ | 272.22571 | *Pinus attenuata*, *P. massoniana*, *P. sativa*, *P. torreyana*, *Pseudotsuga wilsoniana* |
| Abieta-7, 13-diene | C_20_H_32_ | 272.22571 | *Pinus halepensis* |
| (8alpha,13S)-8,13:13,17-Diepoxy-14,15-dinorlabdane, 19-Hydroxy-15,16-dinor-8(17)-labden-13-one | C_18_H_30_O_2_ | 279.16141 | *Pinus halepensis, P. luchuensis* |
| (-)-Maackiain, izalpinin | C_16_H_12_O_5_ | 284.2485 | *Pinus halepensis*, *P. koraiensis*, *P. sylvestris* |
| Flavokawin B | C_17_H_16_O_4_ | 284.2485 | *Pinus cembra*, *P. pentaphylla*, *Pseudotsuga wilsoniana* |
| 3, 5, 7-Trihydroxy-6-methylflavanone, poriol | C_16_H_14_O_5_ | 286.22839 | *Pinus halepensis*, *P. monticola*, *P. ponderosa*, *P. resinosa*, *P. sylvestris*, *Pseudotsuga menziesii*, *P. wilsoniana* |
| Androstenedione | C_19_H_26_O_2_ | 286.22839 | *Pinus morrisonicola*, *Pseudotsuga menziesii* |
| Abieta-7, 13-diene-18-al, pomiferin A, pumiloxide | C_20_H_30_O | 286.22839 | *Pinus halepensis*, *P. kochiana*, *P. radiata*, *P. sylvestris*, *Pseudotsuga wilsoniana* |
| Testosterone | C_19_H_28_O_2_ | 288.23749 | *Pinus pallasiana*, *Pseudotsuga menziesii* |
| Abieta-7, 13-diene-18-ol, elliotinol | C_20_H_32_O | 288.23749 | *Pinus cembra*, *P. halepensis* |
| (-)-Epicatechin, (+)-catechin | C_15_H_14_O_6_ | 290.26889 | *Pinus krempfii*, *P. lambertiana*, *P. pallasiana*, *P. sylvestris*, *Pseudotsuga sinensis* |
| 13-Epimanoyl oxide, epi-13-Manool, Geranyllinalool, Isoabienol, Isocembrol | C_20_H_34_O | 290.26889 | *Pinus ayacahuite, P. cembra, P. glabra, P. halepensis, P. kesiya, P. krempfii*, *P. sibirica*, *P. sosnowskyi*, *P. strobus*, *P. sylvestris* |
| 6-C-Methylkaempferol | C_16_H_12_O_6_ | 300.29129 | *Pinus cembra*, *P. sosnowskyi* |
| (2R)-5, 4′-Dihydroxy-7-methoxy-6-methylflavanone | C_17_H_16_O_5_ | 300.29129 | *Pinus radiata*, *Pseudotsuga wilsoniana* |
| Dehydroabietic acid | C_20_H_28_O_2_ | 300.29129 | *Pinus grandis*, *P. parviflora*, *P. radiata*, *P. sylvestris*, *Pseudotsuga wilsoniana* |
| 5, 7, 3′ 5′-Tetrahydroxy-6-methylflavanone | C_16_H_14_O_6_ | 302.22339 | *Pinus pumila*, *Pseudotsuga wilsoniana* |
| (+)-Pimaric acid, abeoanticopalic acid, abietic acid, cycloanticopalic acid, isopimaric acid, sandaracopimaric acid, trans-communic acid | C_20_H_30_O_2_ | 302.22339 | *Pinus cembra*, *P. contorta*, *P. grandis*, *P. halepensis*, *P. lumholtzii*, *P. montana*, *P. montezumae*, *P. pallasiana*, *P. parviflora*, *P. sativa*, *P. sylvestris*, *P. taeda*, *Pseudotsuga japonica*, *P. menziesii*, *P. wilsoniana* |
| Dihydroquercetin | C_15_H_12_O_7_ | 304.24091 | *Pinus ponderosa*, *Pseudotsuga wilsoniana* |
| Anticopalic acid | C_20_H_32_O_2_ | 304.24091 | *Pinus halepensis*, *P. sylvestris* |
| Catechin-4beta-ol | C_15_H_14_O_7_ | 306.07059 | *Pinus ponderosa, Pseudotsuga wilsoniana* |
| 13-Epitorreferol, 8alpha, 13S-epoxy-14-labden-6alpha-ol, torulosol | C_20_H_34_O_2_ | 306.07059 | *Pinus ayacahuite*, *P. cembra*, *P. luchuensis*, *P. sibirica*, *P. sosnowskyi*, *P. taeda* |
| (2R, 3R)-Pinobanksin 3-acetate, sylpin | C_17_H_14_O_6_ | 315.22211 | *Pinus halepensis*, *P. pallasiana* |
| Pinoquercetin | C_16_H_12_O_7_ | 316.22531 | *Pinus kochiana*, *P. sylvestris* |
| Lambertianic acid | C_20_H_28_O_3_ | 316.22531 | *Pinus glabra*, *P. halepensis*, *P. strobus*, *P. sylvestris* |
| Ampelopsin | C_15_H_12_O_8_ | 320.26099 | *Pinus massoniana*, *P. virginiana* |
| Gibberellin A7 | C_19_H_22_O_5_ | 331.15979 | *Pinus armandii*, *P. ponderosa* |
| Methyl 15-hydroxydehydroabietate, methyl lambertianate | C_21_H_30_O_3_ | 331.15979 | *Pinus grandis*, *P. pumila* |
| Pinomyricetin | C_16_H_12_O_8_ | 332.1622 | *Pinus kochiana*, *P. sylvestris* |
| Gibberellin A4, gibberellin A20 | C_19_H_24_O_5_ | 332.1622 | *Pinus aristata*, *P. nigra*, *P. pumila*, *P. sibirica*, *Pseudotsuga menziesii*, *P. wilsoniana* |
| Gibberellin A12 | C_20_H_28_O_4_ | 332.1622 | *Pinus nigra*, *Pseudotsuga menziesii* |
| Dehydropinifolic acid | C_20_H_30_O_4_ | 334.30731 | *Pinus monticola*, *Pseudotsuga menziesii* |
| 8alpha, 13R-Epoxy-14-labden-19-oic acid | C_21_H_34_O_3_ | 334.30731 | *Pinus monticola*, *Pseudotsuga menziesii* |
| Gibberellin A1, gibberellin A60 | C_19_H_24_O_6_ | 349.20291 | *Pinus koraiensis*, *P. pumila* |
| (-)-Cubebin | C_20_H_20_O_6_ | 356.27219 | *Pinus halepensis*, *P. mugo*, *P. sylvestris*, *Pseudotsuga menziesii* |
| Meridinol | C_20_H_18_O_7_ | 370.27911 | *Pseudotsuga wilsoniana* |
| Sitostenone | C_29_H_48_O | 412.40509 | *Pinus sativum*, *P. sibirica*, *Pseudotsuga wilsoniana* |
| (-)-beta-sitosterol | C_29_H_50_O | 415.3981 | *Pinus parviflora*, *P. rigida* |
| Prunin | C_21_H_22_O_10_ | 434.37119 | *Pinus wallichiana* |
| Massonianoid A | C_26_H_30_O_10_ | 503.43079 | *Pinus halepensis* |
| Melezitose | C_18_H_32_O_16_ | 505.36389 | *Pinus pentaphylla*, *P. ponderosa* |
| Massonianoid A, massonianoside C | C_26_H_34_O_10_ | 507.367 | *Pinus halepensis* |
| Syringetin 3-glucoside | C_23_H_24_O_13_ | 509.36099 | *Pinus excelsa* |
| Massonianoside D | C_25_H_32_O_11_ | 509.36099 | *Pinus grandis* |
