# Supplementary material for: Source identification of western Oregon Douglas-fir wood cores using mass spectrometry and random forest classification
Source: Appl Plant Sci. 2017 May 12;5(5):apps.1600158. doi: 10.3732/apps.1600158 (PMC5435404; doi:10.3732/apps.1600158)

Appendix S5. The purpose of this analysis was to demonstrate that the SOURCE$_{INDIV}$ model shows the same classification accuracy as the SOURCE$_{MEAN}$ model when the sample sizes used to train the models are equivalent. All models were trained in the same manner, see Methods, main text.

Table S5.1. Results of the random forest classification analysis for each model. The estimated mean classification accuracies after 500 iterations are listed for either randomized or observed data and 95% confidence intervals are in parentheses. Estimated mean classification accuracy is the complement of the estimated mean of the median out-of-bag classification error for 500 iterations.

Model	Sample size	Randomized	Observed
SOURCE$_{INDIV}$	560	49.8%	75.7%
		(49.5, 50.0)	(75.6, 75.8)
SOURCE$_{INDIV}$	188	48.8%	71.9%
		(48.4, 49.2)	(71.6, 72.2)
SOURCE$_{MEAN}$	188	48.9%	70.1%
		(48.5, 49.3)	(70.0, 70.2)

Fig. S5.1. Distributions of classification accuracies for the SOURCE$_{INDIV}$ model with 560 samples corresponding to 560 annual rings (outlined in blue), the SOURCE$_{INDIV}$ model with 188 samples corresponding to 188 annual rings (outlined in gold), and the SOURCE$_{MEAN}$ model with 188 samples corresponding to 188 mass spectra averaged over three annual rings or growth years (outlined in red). Dark gray distributions were generated from randomized data, and light gray distributions were generated from observed data. Blue lines indicate the estimated mean classification accuracy across the three models for observed data, and black lines indicate the estimated mean classification accuracy across the three models for randomized data. 95% confidence intervals are listed in Table S5.1.

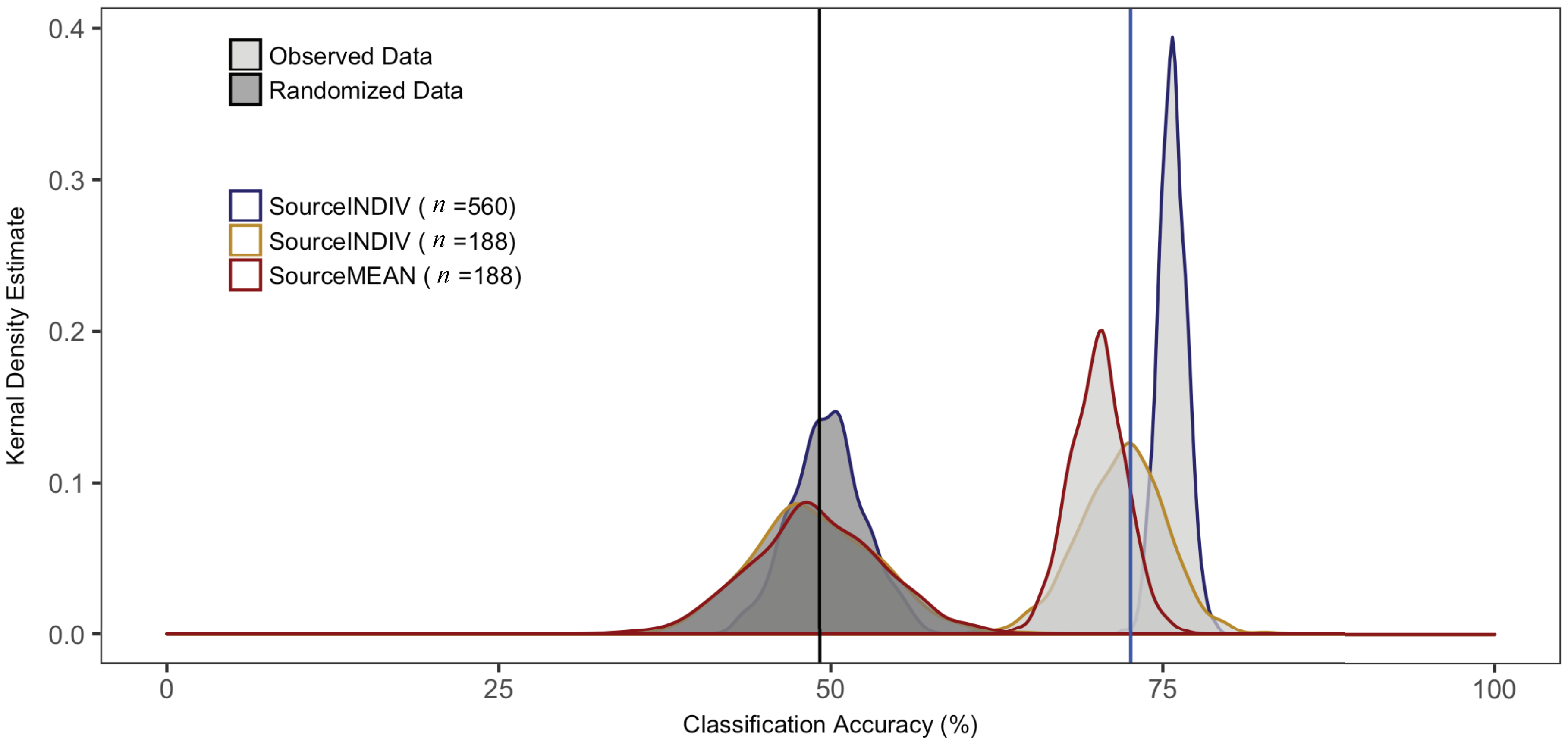

Fig. S5.2. ROC curves generated for 500 random forests by predicting the class membership of each sample in a validation set. The *x*-axis is the false-positive rate and the *y*-axis is the true-positive rate. Gray lines indicate individual ROC curves from each of the 500 iterations. Colored lines indicate the estimated mean ROC curve generated with a generalized additive model and a cubic spline. (A) ROC plots for the $SOURCE_{INDIV}$ model with 560 samples corresponding to 560 annual rings, (B) ROC plots for the $SOURCE_{MEAN}$ model 188 samples corresponding to 188 mass spectra averaged over three annual rings or growth years, (C) ROC plots for the $SOURCE_{INDIV}$ model with 188 samples corresponding to 188 annual rings, and (D) superimposed mean ROC curves for the $SOURCE_{INDIV}$ model (n = 560; blue), the $SOURCE_{INDIV}$ model (n = 188; gold), and the $SOURCE_{MEAN}$ model (n = 188; red).

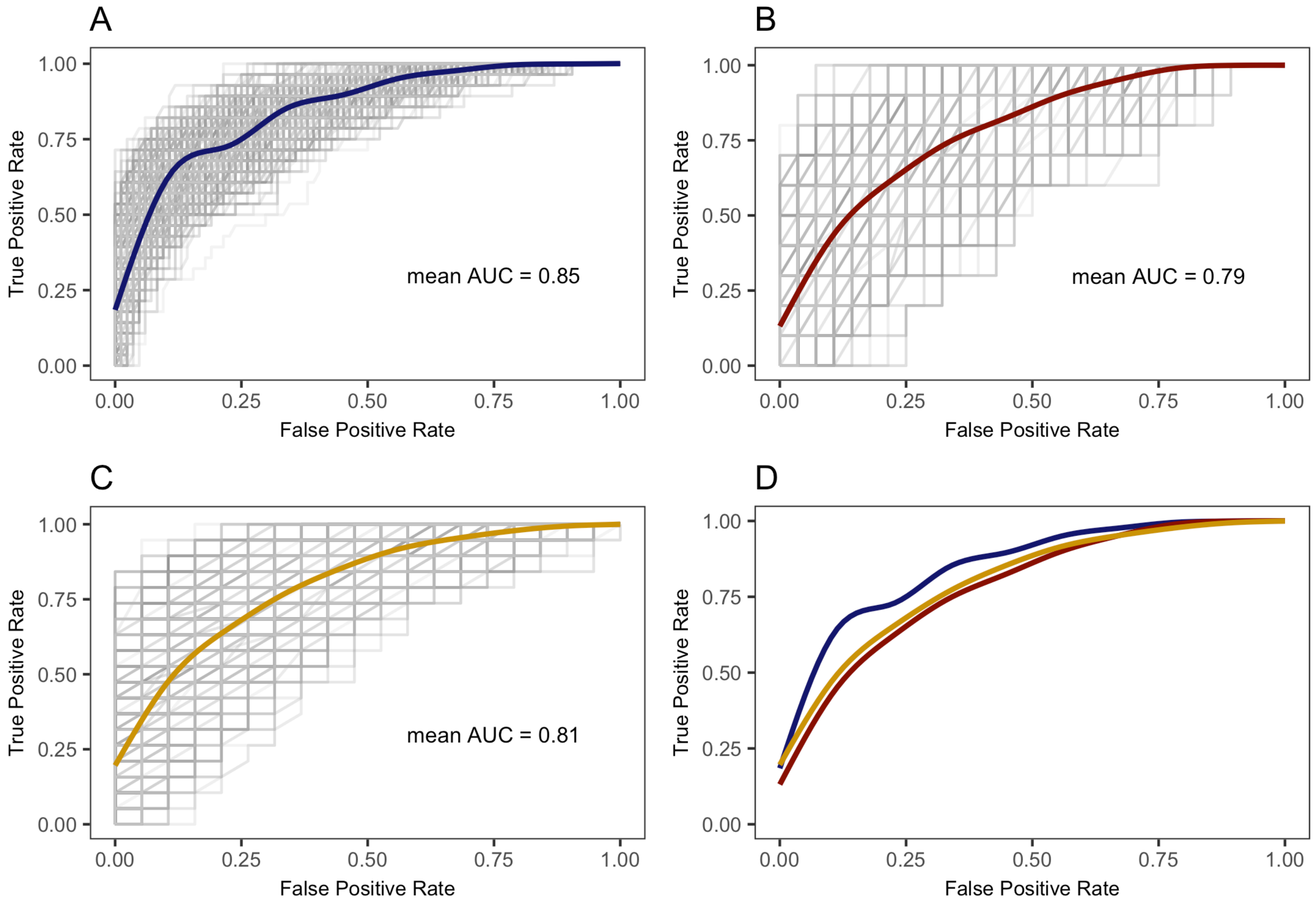

Supplement: Supplementary file 5 [file apps.1600158_s5.pdf]
