# Supplementary material for: Source identification of western Oregon Douglas-fir wood cores using mass spectrometry and random forest classification
Source: Appl Plant Sci. 2017 May 12;5(5):apps.1600158. doi: 10.3732/apps.1600158 (PMC5435404; doi:10.3732/apps.1600158)

APPENDIX S6. The purpose of this analysis was to demonstrate that classification accuracy for the SOURCE$_{INDIV}$ model and the SOURCE$_{MEAN}$ model can be improved by using only the 50 highest-scoring molecules on the Gini impurity index. The analysis of the main text is effectively a "first pass" analysis, and using only high Gini molecules will better optimize the models. Models were trained in the same manner as described in the Methods (main text).

Table S6.1. Results of the random forest classification analysis for each model with a reduced data set (high Gini molecules only). The estimated mean classification accuracies after 500 iterations are listed for either randomized or observed data and 95% confidence intervals are in parentheses. Estimated mean classification accuracy is the complement of the estimated mean of the median out-of-bag classification error for 500 iterations. Compare to Table 2 (main text).

Model	Sample size	Randomized	Observed
SOURCE$_{INDIV}$	560	49.8%	76.8%
		(49.6, 50.1)	(76.7, 76.9)
SOURCE$_{MEAN}$	188	49.0%	74.1%
		(48.6, 49.5)	(74.0, 74.2)

Fig. S6.1. Distributions of the classification accuracies from random forests generated with reduced data sets (highest Gini). Dark gray distributions were generated from randomized data, and light gray distributions were generated from observed data. Blue lines indicate the estimated mean classification accuracy for observed data, and black lines indicate the estimated mean classification accuracy for randomized data. 95% confidence intervals are listed in Table S6.1. Classification accuracies are shown for (A) SOURCE$_{INDIV}$ and (B) SOURCE$_{MEAN}$ models. Compare to Fig. 3 (main text).

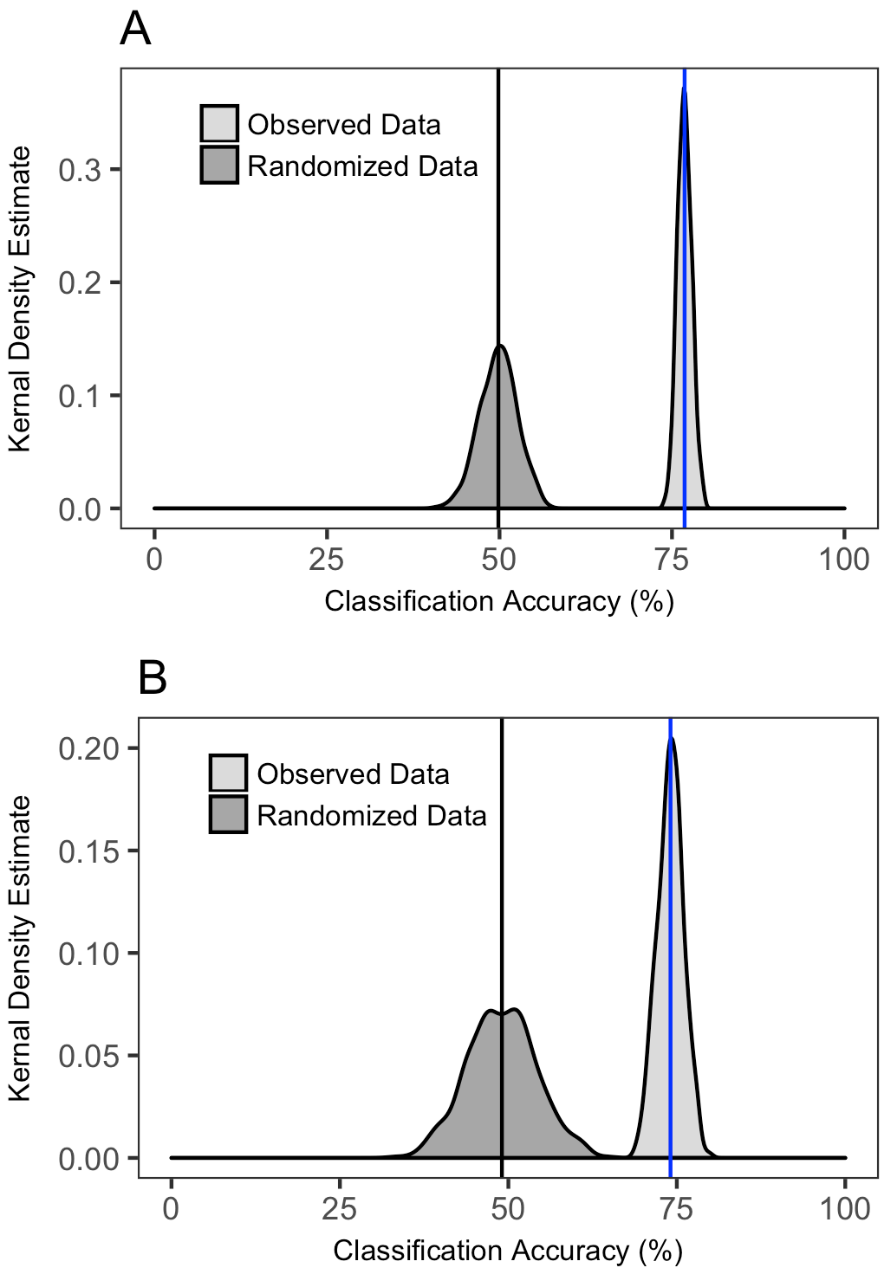

Fig. S6.2. ROC curves generated for 500 random forests by predicting the class membership of each sample in a validation set. The *x*-axis is the false-positive rate and the *y*-axis is the true-positive rate. Gray lines indicate individual ROC curves from each of the 500 iterations. Colored lines indicate the estimated mean ROC curve generated with a generalized additive model and a cubic spline. (A) ROC plots for the SOURCE$_{INDIV}$ model with only the 50 highest-ranking molecules according to the Gini index, (B) ROC plots for the SOURCE$_{MEAN}$ model with only the 50 highest-ranking molecules according to the Gini index, and (C) superimposed mean ROC curves for the SOURCE$_{INDIV}$ model (50 highest Gini; blue) and the SOURCE$_{MEAN}$ model (50 highest Gini; red). Compare to Fig. 4 (main text).

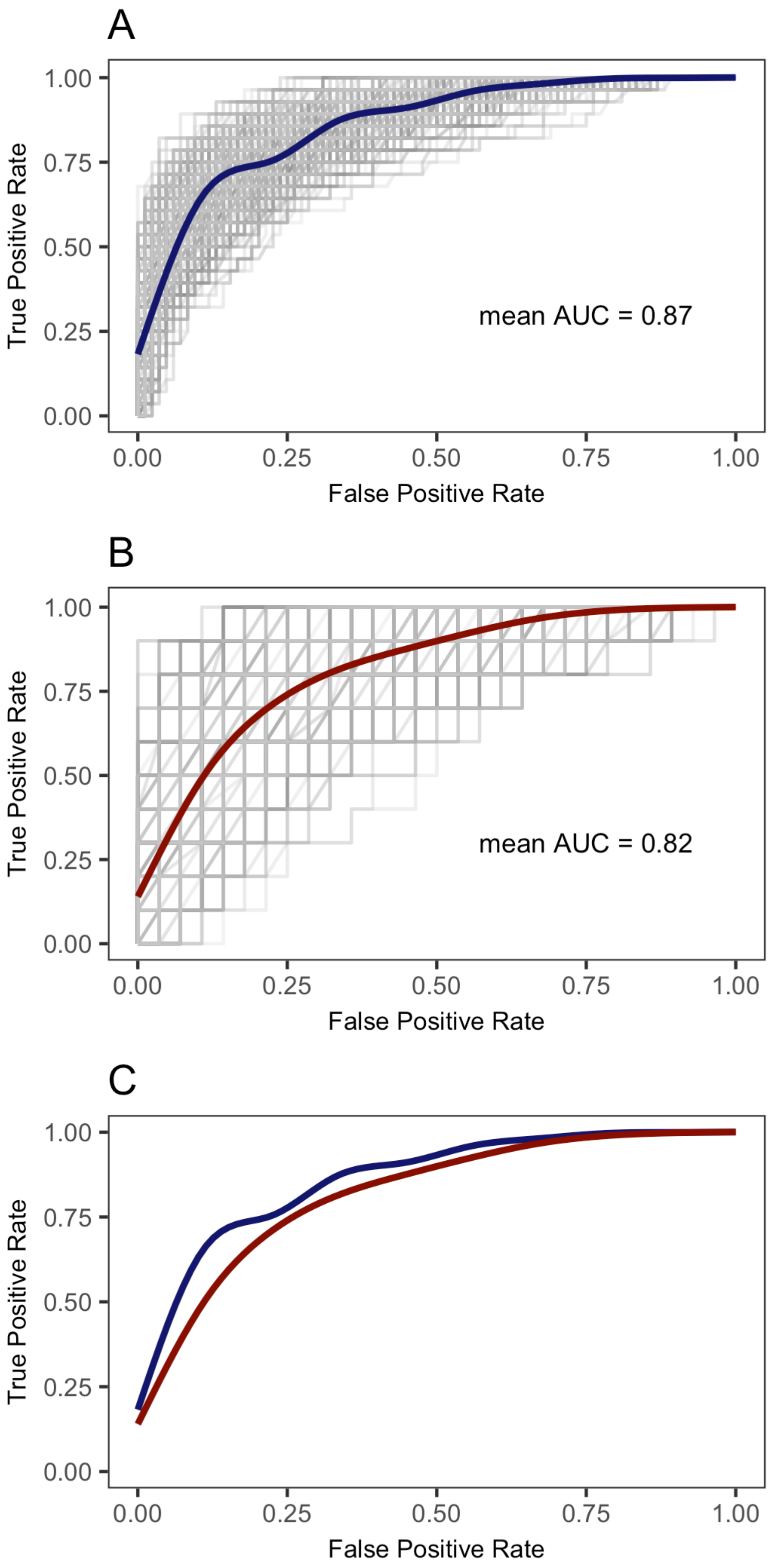

Supplement: Supplementary file 6 [file apps.1600158_s6.pdf]
