# Supplementary material for: Source identification of western Oregon Douglas-fir wood cores using mass spectrometry and random forest classification
Source: Appl Plant Sci. 2017 May 12;5(5):apps.1600158. doi: 10.3732/apps.1600158 (PMC5435404; doi:10.3732/apps.1600158)
Supplement: Supplementary file 7 [file apps.1600158_s7.pdf]

APPENDIX S7. Methods and results from our linear discriminant analysis/leave-one-out cross validation classification analysis.

MATERIALS AND METHODS

Samples**—**Refer to main text.

Mass spectrometry**—**Refer to main text.

Data analysis**—**Analyses were conducted using TSSPro3 (Shrader Analytical Labs, Detroit, Michigan, USA), Mass Mountaineer (RBC Software, Peabody, Massachusetts, USA), and R version 3.3.2 (R Core Team, 2016) with the package ggplot2 (Wickham, 2009). We used TSSPro3 processing software to obtain mass spectra corresponding to: (1) each annual ring analyzed via DART-TOFMS (three mass spectra per individual; $n = 560$), and (2) a mass spectrum averaged over growth years 1986–1988 (one mass spectrum per individual; $n = 188$). Mass spectra include estimated mass-to-charge ratios (m/z) and relative molecule abundance (0–100%), with each molecule relativized to the molecule in the mass spectrum with the highest abundance (Cody, 2015).

We performed linear discriminant analysis (LDA) in Mass Mountaineer for each of our models (Table 1, main text) using 38 diagnostic molecules from a reference sample mass spectrum, which was a single annual ring corresponding to growth year 1988 from a Cascade Range tree (44.60163°N, 121.95015°W). We selected a mass tolerance of 250 mDa for the pre-selected diagnostic molecules and a minimum relative abundance of 1%. We used Mass Mountaineer and a list of publicly available molecules from the genus *Pseudotsuga* and related *Pinus* to tentatively identify the 38 diagnostic molecules used for LDA (Shinbo et al., 2006). We calculated the LDA coordinates of each sample in Mass Mountaineer and plotted the LDA ordinations. Class prediction accuracy for LDA was assessed with leave-one-out cross-validation (LOOCV), an algorithmic process that iteratively treats each mass spectrum as an unknown and assigns it to a class in the grouping variable (Xi et al., 2014). LOOCV accuracies range from 100% (all sample mass spectra were correctly assigned to class) to 1/N%, where N is the number of classification classes (e.g., 50% for two classes).

To evaluate whether LDA-based LOOCV accuracy was higher than random classification, we performed randomization tests to determine the expected random classification accuracy for LDA. Randomizations were performed by randomly selecting mass spectra and assigning them to a class in the grouping variable. We used 20 iterations because random selection of mass spectra and input into Mass Mountaineer is a manual process.

RESULTS

Linear discriminant analysis—Our analysis evaluated the suitability of four classification models for Douglas-fir wood metabolites, including SOURCE$_{INDIV}$, SOURCE$_{MEAN}$, YEAR, and YEAR*SOURCE (Table 1, main text). The results from these analyses are summarized in Table S7.1 and described below.

Table S7.1. Results of the LDA/LOOCV classification analysis for each model with randomized and observed data. The LDA/LOOCV classification accuracy for observed data and the estimated mean LDA/LOOCV classification accuracy for randomized data are listed. For randomized data, 95% confidence intervals were calculated after 20 iterations of each model and are listed in parentheses.

Model identifier	Classes	Random	Observed
SOURCE$_{INDIV}$	2	42.9%	72.9%
(95% CI)		(41.6, 44.2)	(—)
SOURCE$_{MEAN}$	2	46.1%	72.9%
		(43.7, 48.5)	(—)
YEAR	3	28.3%	30.4%
		(27.2, 29.4)	(—)
YEAR*SOURCE	6	14.4%	20.4%
		(13.5, 15.4)	(—)

SOURCE$_{INDIV}$ model—This model tested classification accuracy to geographic source variation in wood chemistry. All individual annual rings were classified to one of two location categories (Coast, Cascades). For this test, we treated the time series samples (1986–1988) from a single tree as independent replicates, even though they are not independent. The LDA density plot showed separation of the sample mass spectra according to source region (Fig. S7.1A). We plotted a single axis, which captured nearly 100% of total variation among sample mass spectra. The LDA-based LOOCV classification accuracy for these samples was 72.9% (Table S7.1; Fig. S7.2A, blue line), which was significantly higher than the estimated mean LOOCV classification accuracy from 20 randomizations (42.9%; Table S7.1; Fig. S7.2A, black line).

SOURCE$_{MEAN}$ model—This model also tested classification accuracy to geographic source variation in wood chemistry. Mean spectral abundance values for samples were classified to one of two location categories (Coast, Cascades). The mean value from three, time-series samples (1986–1988) was evaluated, and the means represent independent estimates. Similar to the classification of individual samples, this model separated the mean sample mass spectra according to source region (Fig. S7.1B). The LDA-based LOOCV classification accuracy for these samples was 72.9% (Table S7.1; Fig. S7.2B, blue line), which was significantly higher than the estimated mean LOOCV classification accuracy from 20 randomizations (46.3%; Table S7.1; Fig. S7.2B, black line).

YEAR model—The LDA ordination based on 38 diagnostic molecules showed little to no discrimination of sample mass spectra according to growth year (Fig. S7.1C), with the first linear discriminant axis (LD 1) capturing 68.5% of total inter-annual variation among sample mass spectra. The LDA-based LOOCV classification accuracy was estimated to be 30.4% (Table S7.1; Fig. S7.2C, blue line), and this value was nearly equivalent to the value obtained from 20 randomized sample mass spectra (28.3%; Table S7.1; Fig. S7.2C, black line).

Fig. S7.1. (A) LDA for the SOURCE$_{INDIV}$ model. (B) LDA for the SOURCE$_{MEAN}$ model. (C) LDA for the YEAR model. Gray points, gold points, and purple points correspond to growth years 1986, 1987, and 1988 respectively. (D) LDA for the YEAR*SOURCE model. Light red points, red points, and dark red points correspond to the Cascade Range and growth years 1986, 1987, and 1988, respectively. Light blue points, blue points, and dark blue points correspond to the Coast Range and growth years 1986, 1987, and 1988, respectively.

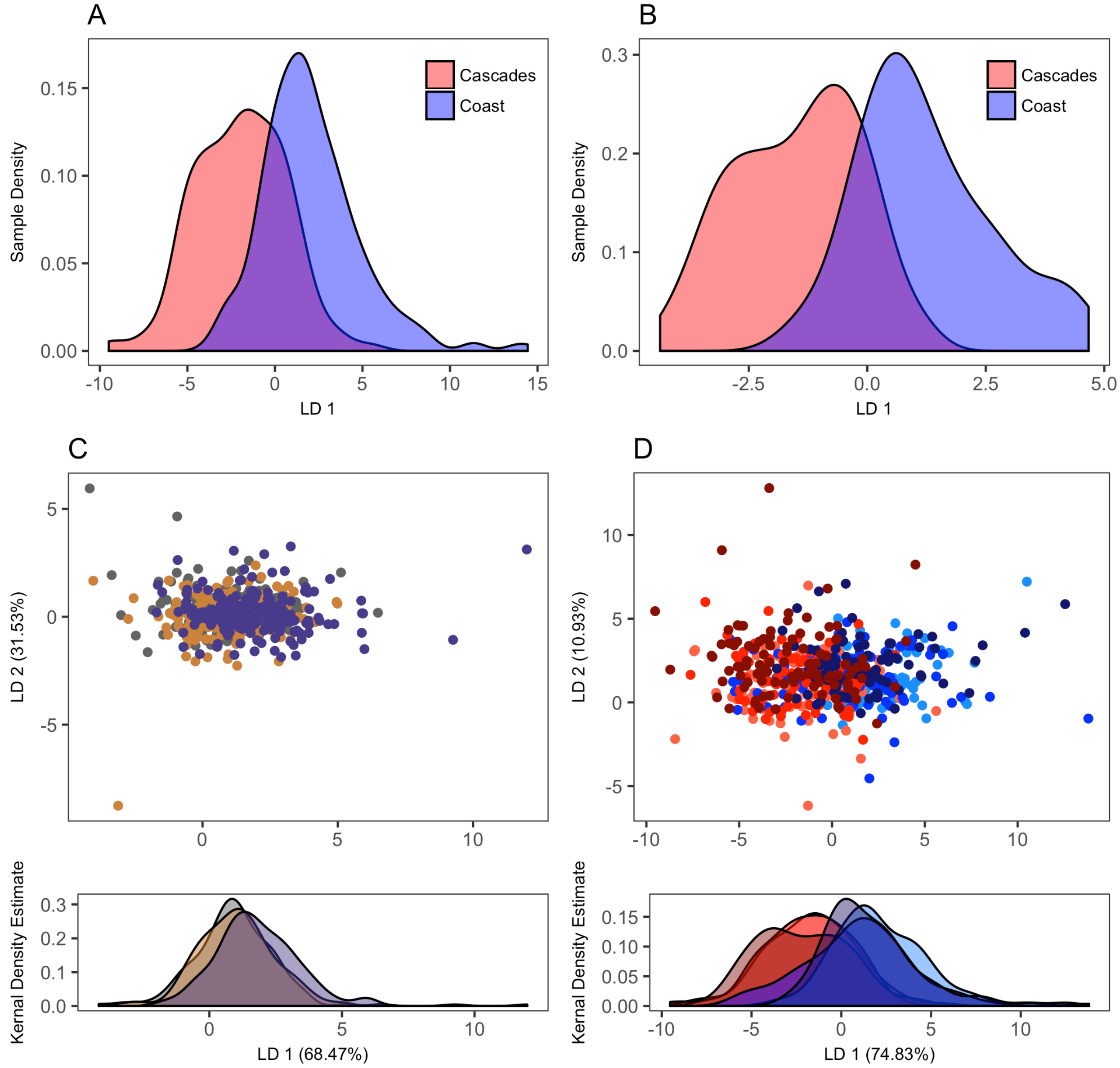

Fig. S7.2. Comparison of LOOCV classification accuracies for observed data (blue lines) and distributions of LOOCV classification accuracies generated with randomized data (gray density plots). The black lines indicate the estimated mean LOOCV classification accuracy for LDAs built with randomized data. LDA/LOOCV classification accuracy distribution with randomized data and the LOOCV classification accuracy with observed data for (A) the SOURCE$_{INDIV}$ model, (B) the SOURCE$_{MEAN}$ model, (C) YEAR model, and (D) the YEAR*SOURCE model.

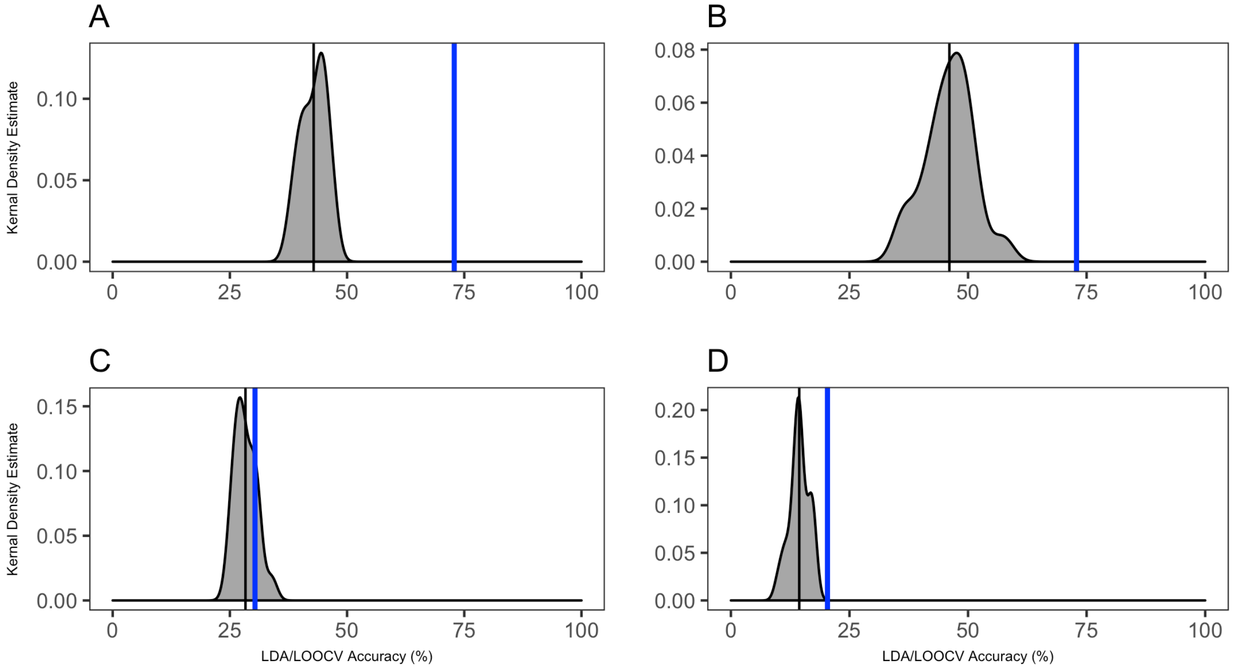

*YEAR*SOURCE model*—The LDA showed little to no discrimination of sample mass spectra according to growth year, but the first axis (Linear Discriminant Score 1) captured 74.9% of variation among sample mass spectra. LD1 was interpreted as capturing geographic variation (Fig. S7.1D) because of the observed separation by source (Cascades [red] or Coast [blue]). The LOOCV classification accuracy for this model was estimated at 20.4% (Table S7.1; Fig. S7.2D, blue line). The LDA-based LOOCV classification accuracy of 20 randomized spectra was 14.4% (Table S7.1; Fig. S7.2D, black line).

Molecule importance—Using Mass Mountaineer, we inferred the identity of 20 of the 38 diagnostic molecules use for LDA (Table S7.2).

Table S7.2. Putative identities for 20 of the 38 diagnostic molecules used for our LDA/LOOCV classification analysis. Identities were approximated in Mass Mountaineer by comparing the mass-to-charge ratio of each molecule to a list of molecules identified in *Pinus* and *Pseudotsuga*. Provided are names that have been used to describe the molecules, their molecular formula, their mass-to-charge ratio, and the species from which they were identified.

Molecule name	Molecular formula	Mass (*m/z*)	Species
2-Phenylethanol	$C_8H_{10}O$	123.1165	*Pinus* spp.
Estragol	$C_{10}H_{12}O$	149.13229	*Pinus sylvestris*
Indole-3-ethanol	$C_{10}H_{11}NO$	161.1317	*Pinus sylvestris*
Indole-3-carboxylic acid	$C_9H_7NO_2$	161.1317	*Pinus sylvestris*
Methyl indole-3-acetate	$C_{11}H_{11}NO_2$	189.1624	*Pinus contorta*, *P. sylvestris*, *Pseudotsuga menziesii*
N6-(delta2-Isopentenyl)adenine	$C_{10}H_{13}N_5$	203.1801	*Pseudotsuga menziesii*
(R)-(-)-alpha-Curcumene	$C_{15}H_{22}$	203.1801	*Pinus halepensis*
(-)-Germacrene D, (-)-Isocaryophyllene, (-)-Zingiberene, (E)-beta-Bourbonene, (E)-Caryophyllene, (Z)-beta-Farnesene, alpha-Muurolene, beta-Gurjunene, beta-Sesquiphellandrene, Copaene, Cyclohexane, delta-Cadinene, gamma-Cadinene, gamma-Muurolene, Humulene, Longicyclene, Longifolene	$C_{15}H_{24}$	205.19569	*Pinus eldarica*, *P. halepensis, P. kochiana, P. longifolia, P. pallasiana, P. palustris, P. sosnowskyi, P. sylvestris, P. thunbergii, Pseudotsuga japonica*
Omega-Hydroxydodecanoic acid	$C_{12}H_{24}O_3$	217.1974	*Pinus radiata*
Chrysin	$C_{15}H_{10}O_4$	255.21429	*Pinus aristata, P. excelsa, P. monticola, Pseudotsuga wilsoniana*
Pinocembrin	$C_{15}H_{12}O_4$	257.22919	*Pinus cembra,*

			Pseudotsuga wilsoniana
(2S)-Pinocembrin, 8-Methylpinocembrin, Strobopinin	$C_{16}H_{14}O_4$	271.241	*Pinus krempfii, P. strobus, Pseudotsuga wilsoniana*
bietatriene	$C_{20}H_{30}$	271.241	*Pinus pallasiana*
Naringenin, Pinobanksin	$C_{15}H_{12}O_5$	272.2486	*Pinus banksiana, Pseudotsuga wilsoniana*
Abieta-7, 13-diene	$C_{20}H_{32}$	272.2486	*Pinus contorta, P. grandis*
(-)-Maackiain, Izalpinin	$C_{16}H_{12}O_5$	285.22281	*Pinus morrisonicola, P. sativa*
Flavokawin B	$C_{17}H_{16}O_4$	285.22281	*Pinus excelsa, P. wallichiana*
3,5,7-Trihydroxy-6-methylflavanone, Poriol	$C_{16}H_{14}O_5$	287.23401	*Pinus lambertiana, P. strobus, Pseudotsuga menziesii, P. wilsoniana*
Androstenedione	$C_{19}H_{26}O_2$	287.23401	*Pinus sylvestris*
Abieta-7, 13-diene-18-al, Pomiferin A, Pumiloxide	$C_{20}H_{30}O$	287.23401	*Pinus contorta, P. grandis, P. pumila, Pseudotsuga wilsoniana*
Testosterone	$C_{19}H_{28}O_2$	288.24139	*Pinus sylvestris*
(+)-Catechin, (-)-Epicatechin	$C_{15}H_{14}O_6$	290.25751	*Pinus sibirica, P. sylvestris*
13-Epimanoyl oxide, epi-13-Manool, Geranyllinalool, Isoabienol, Isocembrol	$C_{20}H_{34}O$	290.25751	*Pinus banksiana, P. contorta, P. koraiensis, P. nigra, P. pinaster, P. sibirica*
6-C-Methylkaempferol	$C_{16}H_{12}O_6$	301.2186	*Pinus contorta*
(2R)-5,4′-Dihydroxy-7-methoxy-6-methylflavanone	$C_{17}H_{16}O_5$	301.2186	*Pseudotsuga wilsoniana*
Dehydroabietic acid	$C_{20}H_{28}O_2$	301.2186	*Pinus luchuensis, P. taeda, Pseudotsuga wilsoniana*
5,7,3′,5′-Tetrahydroxy-6-methylflavanone	$C_{16}H_{14}O_6$	302.22549	*Pseudotsuga sinensis*

(+)-Pimaric acid, Abeoanticopalic acid, Abietic acid, Anticopalic acid, Cycloanticopalic acid, Isopimaric acid, Sandaracopimaric acid, trans-Communic acid	$C_{20}H_{30}O_2$	302.22549	*Pinus contorta, P. elliottii, P. grandis, P. luchuensis, P. massoniana, P. palustris, P. strobus, P. sylvestris, P. taeda, Pseudotsuga wilsoniana*
Dihydroquercetin	$C_{15}H_{12}O_7$	304.2355	*Pseudotsuga menziesii*
Anticopalic acid	$C_{20}H_{32}O_2$	304.2355	*Pinus monticola*
Catechin-4beta-ol	$C_{15}H_{14}O_7$	307.2637	*Pseudotsuga menziesii*
13-Epitorreferol, 8alpha, 13S-Epoxy-14-labden-6alpha-ol, Torulosol	$C_{20}H_{34}O_2$	307.2637	*Pinus banksiana, P. contorta*
Pinoquercetin	$C_{16}H_{12}O_7$	317.21561	*Pinus ponderosa*
Lambertianic acid	$C_{20}H_{28}O_3$	317.21561	*Pinus koraiensis, P. lambertiana*
